# Metabolism, metabolites, and macrophages in cancer

**DOI:** 10.1186/s13045-023-01478-6

**Published:** 2023-07-25

**Authors:** Mengyuan Li, Yuhan Yang, Liting Xiong, Ping Jiang, Junjie Wang, Chunxiao Li

**Affiliations:** 1 Department of Radiation Oncology, Peking University Third Hospital, Beijing, 100191 China; 2 Institute of Medical Technology, Peking University Health Science Center, Beijing, 100191 China

**Keywords:** Tumour-associated macrophages, Metabolism, Tumour microenvironment, Metabolism reprogramming, Cancer

## Abstract

Tumour-associated macrophages (TAMs) are crucial components of the tumour microenvironment and play a significant role in tumour development and drug resistance by creating an immunosuppressive microenvironment. Macrophages are essential components of both the innate and adaptive immune systems and contribute to pathogen resistance and the regulation of organism homeostasis. Macrophage function and polarization are closely linked to altered metabolism. Generally, M1 macrophages rely primarily on aerobic glycolysis, whereas M2 macrophages depend on oxidative metabolism. Metabolic studies have revealed that the metabolic signature of TAMs and metabolites in the tumour microenvironment regulate the function and polarization of TAMs. However, the precise effects of metabolic reprogramming on tumours and TAMs remain incompletely understood. In this review, we discuss the impact of metabolic pathways on macrophage function and polarization as well as potential strategies for reprogramming macrophage metabolism in cancer treatment.

## Introduction

**Fig. 1:**
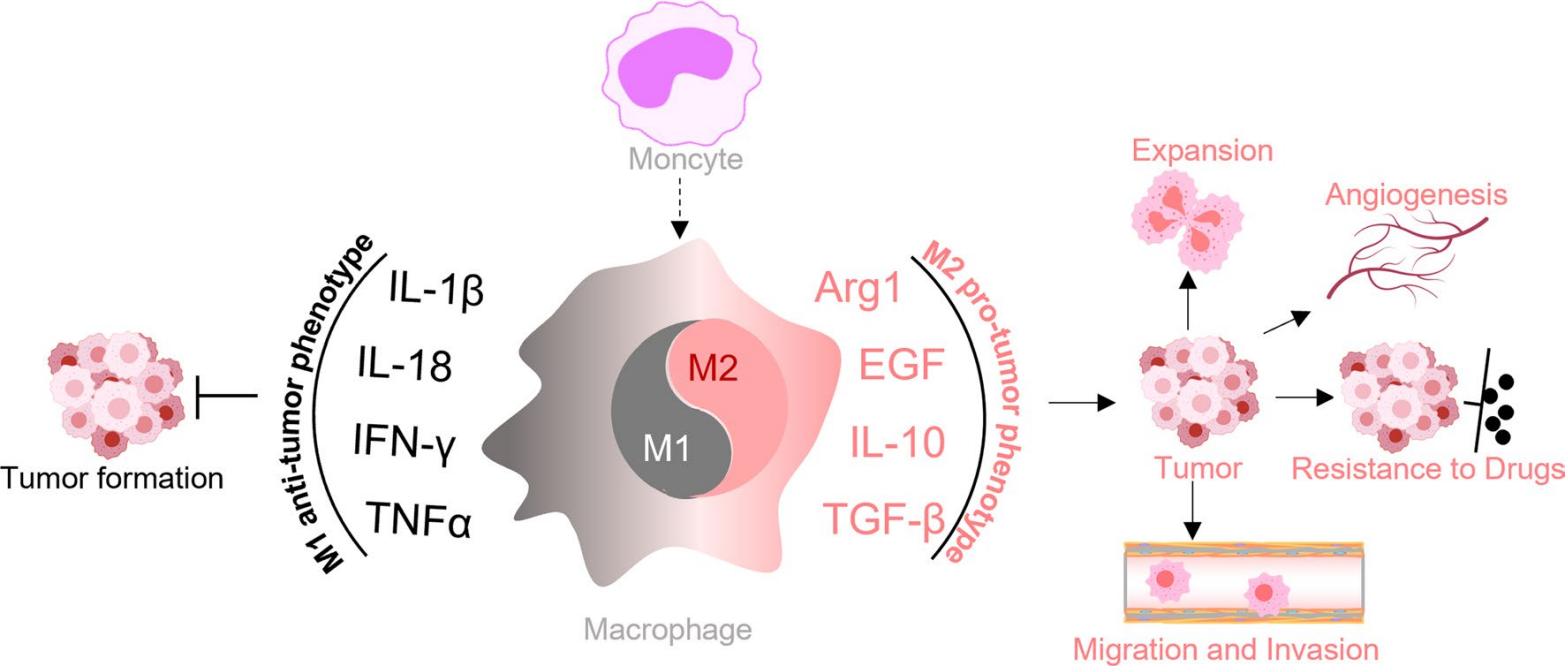
Macrophage polarization and its function in cancer progression. After differentiation into macrophages from monocytes, macrophages can be further polarized into M1 and M2 subsets under different stimuli or microenvironments. M1 macrophages inhibit tumourigenesis by secreting IL-1β, IL-18, IFN-γ, and TNF-α, whereas M2 macrophages promote cancer development through several biological molecules, such as Arg1, IL-4, and TGF-β

Macrophages are crucial immune cells in the body and play essential roles in both innate and adaptive immune responses [1]. Along with other phagocytes, they form the initial line of defence by releasing proinflammatory cytokines, which contribute to the activation of the innate immune system and subsequent T- and B-cell responses [2]. Macrophages can be polarized to acquire different phenotypes based on various stimuli. In the typical classification, proinflammatory macrophages acquire the M1 phenotype induced via lipopolysaccharide (LPS) and anti-inflammatory macrophages acquire the M2 phenotype induced via IL-4 or IL-13 [3, 4] (Table 1). In addition to the M2a phenotype induced by IL-4/IL-13, the M2 subset of macrophages can be further subclassified into different subtypes based on their specific functions. One of these subtypes consists of M2b macrophages, also known as regulatory macrophages. They are activated by immune complexes and TLR ligands and are involved in immune and inflammatory reactions, producing both pro- and anti-inflammatory cytokines. Another subtype consists of M2c macrophages, which are activated by glucocorticoids or IL-10 and primarily exert anti-inflammatory functions. M2d macrophages, also known as TAMs, are activated by TLR ligands and A2 adenosine receptor agonists, and they play crucial roles in regulating tumour progression, angiogenesis and metastasis [4–7]. In addition to the classical categorization of macrophages, there have been several other proposed classification systems based on different criteria. For example, Mosser et al. introduced a new classification method that categorizes macrophages into classically activated macrophages, wound-healing macrophages, and regulatory macrophages based on their homeostatic activities, namely, host defence, wound healing, and immune regulation, respectively [8]. Macrophages within the tumour microenvironment (TME) are referred to as TAMs. As important immune cells that infiltrate the TME, TAMs are characterized by phenotypic plasticity and heterogeneity. Studies have confirmed that TAM heterogeneity is exhibited not only in different cancer patients but also in types of different cancers within the same patient, as well as in different stages of tumour development [9–11]. This heterogeneity reflects the ability of TAMs to respond to environmental stimuli, leading to polarization into phenotypes ranging from a proinflammatory (M1-like) to an anti-inflammatory (M2-like) types [12, 13]. Thus, TAM subsets exert diverse effects on tumourigenesis and tumour progression. For example, during the initiation stages of tumour formation, TAMs mainly play a proinflammatory role and suppress tumour development, although the evidence is still limited [12]. As a tumour grows, macrophages in the TME are “educated” and acquire an M2-like phenotype through the action of Th2 cells. These cytotoxic macrophages then transition into tumour-supporting macrophages, promoting tumour progression [14]. Additionally, specific subsets of TAMs have been associated with various processes such as oncogenesis, angiogenesis, vascularization, immunosuppression, metastasis, resistance to therapy, and poorer clinical outcome [15–17]. However, TAMs can also exhibit tumouricidal functions by mediating tumour phagocytosis and promoting anti-tumour immunity [18, 19]. Notably, TAMs can play dual roles depending on the context. While they can exert a tumour-promoting effect and be associated with poor prognosis in certain cancers, such as breast cancer [20], lung cancer [21], and pancreatic cancer [22], they can also exert anti-tumour effects on colon cancer [23]. Furthermore, the localization of TAMs within the TME influences their functions. Generally, TAMs located in hypoxic areas or close proximity to blood vessels exert proangiogenic effects. On the other hand, TAMs that infiltrate the tumour front have been found to play an anti-tumourigenic role specifically in colon cancer (Fig. 1) [23, 24].

**Table 1:** Metabolic pathways in the macrophages

Metabolism pathway	M1 macrophage	M2 macrophage
Glycolysis	Enhanced glycolysis	Glycolysis is crucial for M2 activation
Amino acid metabolism	Upregulated iNOS	Upregulated Arginase-1 activity
PPP	Increased PPP; increased NADH/NAD + ratio	Restricted PPP
OXPHOS	Inhibited OXPHOS	Enhanced OXPHOS
Lipid metabolism	Increased fatty acid synthase	Enhanced FAO
TCA	Disrupted TCA	Elevated TCA
Glutamine metabolism	No	Contributed to M2 activation

After malignant transformation, intratumour angiogenesis is crucial for further tumour progression. In 1971, Folkman et al. initially proposed a correlation between tumour growth and angiogenesis [25]. Initially, tumour angiogenesis was believed to be induced only by tumour cells. However, Staton et al. discovered that TAMs also regulate angiogenesis through factors such as hypoxia inducible factor-1α (HIF-1α), vascular endothelial growth factor (VEGF), and tissue factor (TF) [26, 27]. In addition, other inflammatory cytokines secreted by TAMs have also been found to promote neovascularization through multiple signalling pathways [28]. Furthermore, TAMs have been implicated in facilitating tumour invasion and metastasis [29, 30]. Therefore, the possibility of targeting TAMs has attracted increasing attention in recent years, and TAMs have been depleted using colony-stimulating factor-1 (CSF-1)/CSF-1R inhibitors and by re-educating TAMs towards an M1-like phenotype through signalling pathways involving CD40, CD47, phosphoinositide 3-kinase (PI3K) and toll-like receptor (TLR) [31–35].

In addition to the aforementioned methods, metabolic reprogramming has also been progressively adopted for regulating macrophage polarization [9] (Table 2). Tumour cells exhibit peculiar metabolic processes that support their nutrient and energy requirements. Although metabolic alterations in tumours are widely studied, tumour-specific metabolic characteristics of tumour cells have not been established. The Warburg effect (aerobic glycolysis) is one of the best characterized metabolic changes in tumour cells [36]. Even under aerobic conditions, tumour cells reprogramme glucose metabolism to utilize glycolysis instead of mitochondrial oxidative phosphorylation (OXPHOS) [37]. In the past, researchers thought that glycolysis was the main source of ATP that maintains tumour cell growth [38]. However, in glycolysis, each glucose molecule produces two ATP molecules, while mitochondrial OXPHOS produces approximately 30 ATP molecules [39]. Therefore, aerobic glycolysis is not the main source of ATP synthesis in tumour cells and many tumours obtain energy through glucose oxidation [40–43]. Nevertheless, studies have shown that high glycolytic rates can provide metabolic precursors for biomass production [44, 45]. In aerobic glycolysis, glucose-derived pyruvate is converted to lactate by lactate dehydrogenase (LDH), which allows the regeneration of NADH, which maintains glycolysis [46]. Moreover, other fuels, such as amino acids, fatty acids, and proteins, can also provide energy for tumour growth [44]. This altered tumour cell metabolism has a profound impact on the TME and may significantly affect the metabolism and polarization of TAMs. However, the mechanisms underlying TAM metabolism and polarization remain poorly characterized, and further investigation is needed. Several studies have confirmed that different metabolites promote TAM polarization into different phenotypes with diverse functions [8, 47]. Therefore, clarifying the mechanisms underlying metabolic processes and macrophage polarization and exploring potential strategies targeting TAMs are necessary. This review discusses the cross talk between the factors involved in metabolism and macrophage polarization, and summarizes a viable strategy for targeting TAMs by reprogramming metabolism.

**Table 2:** Metabolites in macrophage metabolic reprogramming

Metabolites	Changes	Changes in macrophage polarization	Functions in environment
Glucose		Increased in M1; Decreased in M2	
Pyruvate	Increased	M2	
Lactate	Increased	M2	Tumour-promoting
Cholesterol	Decreased	M2	Tumour-promoting
Triglyceride	Decreased	M2	Tumour-promoting
Diglyceride	Increased	M2	Tumour-promoting
Free fatty acid	Increased	M2	Tumour-promoting
α-ketoglutarate	Increased	M2	Immunosuppressive
Succinate	Increased	M1	
Citrate	Increased	M1	
Itaconate	Increased	M1	

## Metabolic influences in the TME

Metabolism changes in cells within the TME exert a profound impact not only on tumour progression but also on the functionality of immune cells. Understanding metabolic influences in immune cell function is crucial for the development of novel immunotherapeutic strategies. Obesity has been identified as a risk factor for the development of various types of tumours [48]. Previous studies on obesity and cancer have predominantly focused on the tumours themselves [49, 50]. However, Ringel et al. demonstrated that a high-fat diet (HFD) promotes colorectal tumour growth in a T cell-dependent manner [51]. Mechanistically, tumour cells can increase fatty acid uptake from a HFD and alter fatty acid distribution within tumours, resulting in impaired CD8^+^ T cell infiltration and function. Inhibition of the metabolic reprogramming of tumour cells restores the anti-tumour function of CD8^+^ T cells. In addition to CD8^+^ T cell, metabolism also plays a regulatory role in other immune cells within the TME [52]. For instance, Ma et al. found that bile acid can promote the infiltration of CXCR6^+^ natural killer T (NKT) cells and enhance their anti-tumour activity in liver cancer [53]. Aberrant glycolysis in tumours leads to increased production of lactic acid in the TME. Studies have confirmed that excessive lactic acid disrupts the metabolism of human cytotoxic T lymphocytes (CTLs), inhibiting their proliferation and reducing cytokine production [54]. This phenomenon can be prevented by inhibitors of lactic acid production. Moreover, in a low-glucose environment, T cells are stimulated to differentiate from effector T cells to acquire a Foxp3^+^ regulatory (Treg) phenotype, which exerts a tumour-promoting effect [55]. Additionally, increased glycolytic metabolism in tumour cells promotes the secretion of tumour granulocyte colony-stimulating factor (G-CSF) and granulocyte macrophage colony-stimulating factor (GM-CSF), which further promotes the recruitment of MDSCs and inhibit T cells activity [56].

Recently, immune checkpoint blockades (ICB) have become widely used in immunotherapy for treating malignant tumours. Studies have found that obesity significantly regulates the expression of the immune checkpoint molecules PD-1 and PD-L1 [52]. Moreover, it has been reported that obese or overweight patients with melanoma, non-small cell lung cancer (NSCLC), or renal cell carcinoma show better responses to ICB immunotherapy [57–59]. Therefore, modulating immune cell metabolism or targeting metabolic vulnerabilities in cancer cells may help enhance anti-tumour immune responses and increase the effectiveness of immunotherapies.

## Cross talk between metabolism and macrophage polarization

As an important part of the innate immune system, macrophages exhibited high plasticity and could effectively respond to various stimuli. M1 macrophages are characterized by enhanced glycolysis, high level of glutathione, increased expression of ferritin, elevated expression of cyclooxygenase (COX) 2, low expression of COX1, augmented activity of inducible nitric oxide synthase (iNOS) 2, and decreased activity of arginase 1 (Arg1). However, M2 macrophages are depicted with enhanced fatty acid oxidation (FAO), low expression of ferritin, reduced levels of glutathione, diminished COX2 production, elevated COX1 production, weak iNOS activity, and increased Arg1 activity.

### Glucose metabolism

**Fig. 2:**
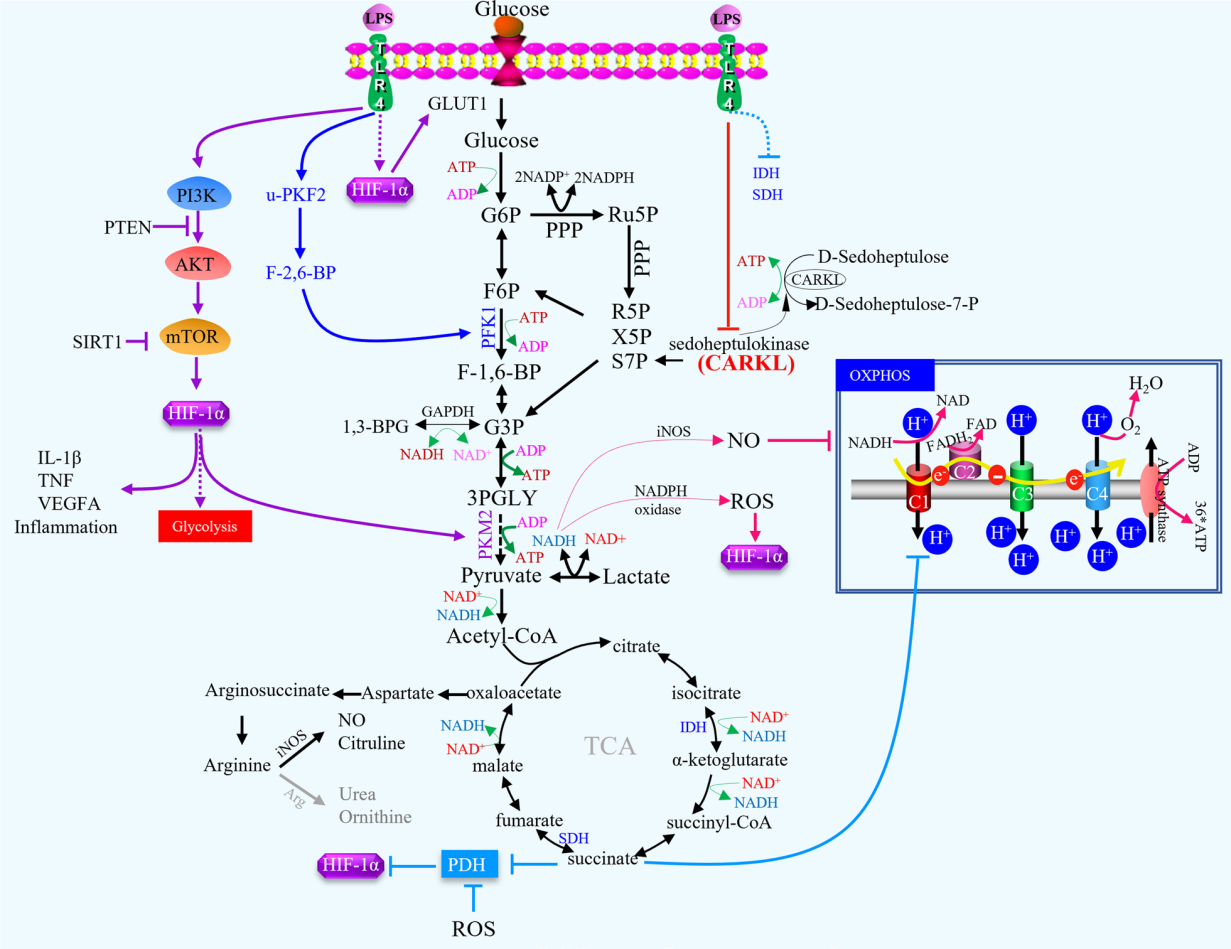
Part of metabolic adaptations of macrophages. Macrophage activation through LPS/IFN-γ results in similar flux distribution patterns towards glycolysis regardless of the pathway activated. HIF-1α activation can increase fructose-2,6-bisphosphate concentration and the glycolytic flux. CARKL could antagonize LPS-induced cytokines production. The decrease of OXPHOS induced by LPS leads to the accumulation of intermediate metabolites in the tricarboxylic acid cycle, especially succinic acid. Succinic acid can transfer from mitochondria to intracellular, inhibit the activity of prolyl hydroxylase (PHD) enzyme, and increase HIF-1α by promoting its stability. Notably, mTOR-HIF-1α axis involves in glycolysis in M1-polarized macrophages. In hypoxia state, HIF-1α can promote glycolysis by inducing expression of the related enzymes and transcriptional effectors. Meanwhile, HIF-1α can promote the expression of proinflammatory genes in macrophages

**Fig. 3:**
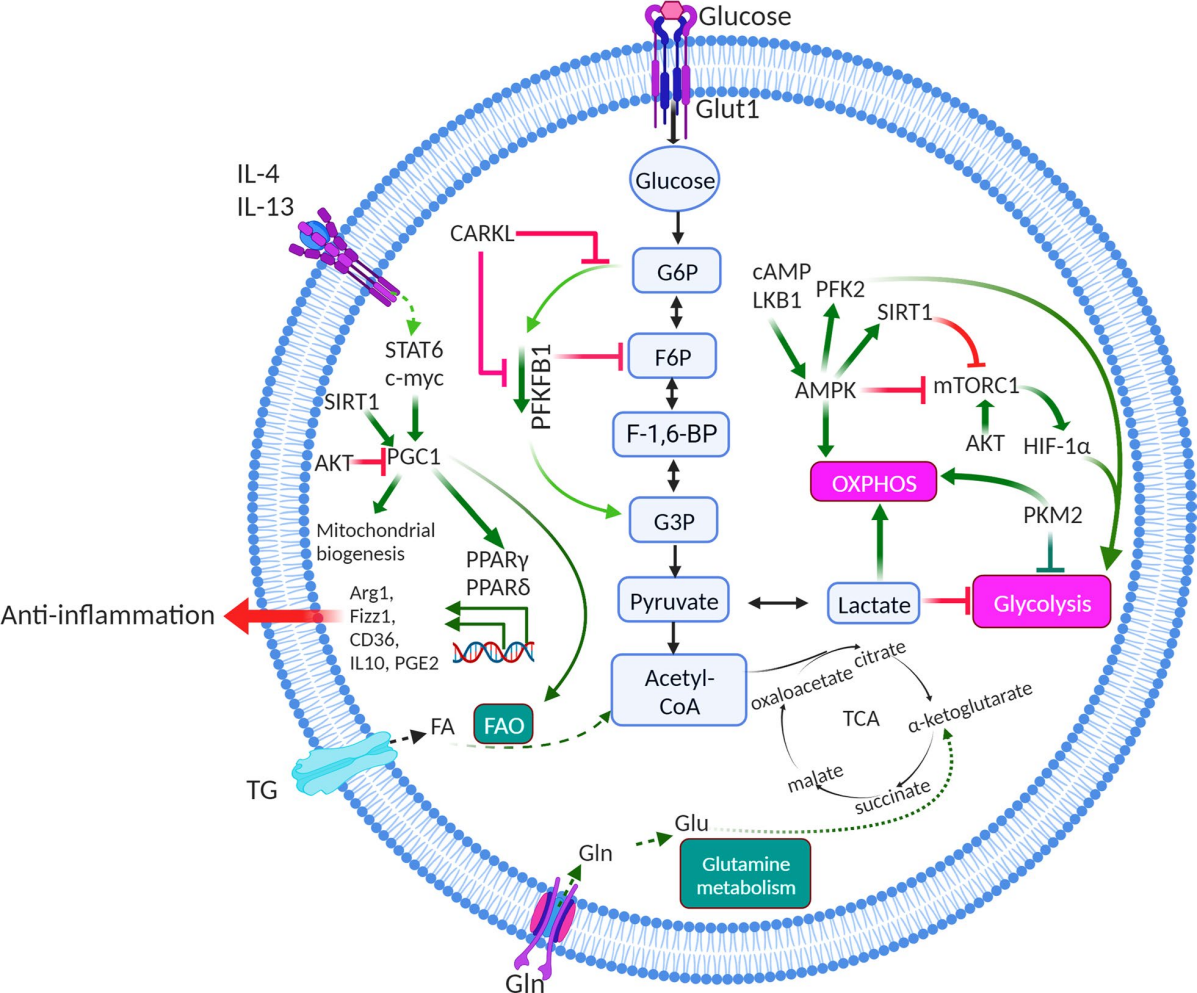
Metabolic reprogramming in macrophages. M1-polarized macrophages primarily depend on glucose and the flux of glucose into lactate, reactive oxygen species (ROS) production, and nitric oxide (NO) generation for tumour killing after stimulation with the cytokines IFN-γ, TNF and LPS, which involves a cell-intrinsic shift towards aerobic glycolysis, generation of ROS, disruption of the TCA cycle, and inhibition of OXPHOS. M2-polarized macrophages primarily depend on β-oxidation of fatty acids and the tricarboxylic acid cycle (TCA cycle) after stimulation of cytokines IL-4, IL-13, and IL-10. During these processes, some key molecules participate in the metabolic mechanisms including mTOR, HIF-1α, SIRT1, and AKT

TAMs can enhance hypoxic and aerobic glycolysis in mouse subcutaneous tumours and in patients with NSCLC by secreting tumour necrosis factor-alpha (TNF-α), whereas increased AMP-activated protein kinase (AMPK) and peroxisome proliferator-activated receptor gamma (PPARγ) co-activator 1-α levels in TAMs facilitate tumour hypoxia [9, 60]. Macrophage polarization is typically associated with glucose metabolism. Activated macrophages are essentially glycolytic cells, with a clear cut-off between classic activation and the alternative pathway. Interestingly, M1 macrophage activation through LPS/IFN-γ, listeria monocytogenes, thioglycolate, TLR-2, -3, -4, or -9 resulted in similar flux distribution patterns towards anaerobic glycolysis, regardless of the activated pathway [61, 62](Fig. 2). However, stimulation via alternative pathways has minor metabolic effects. It has been observed in animal models that the molecular basis of the differences between these two types of behaviour involves a switch in the expression of 6-phosphofructo-2-kinase/fructose-2,6-bisphosphatase (PFK2) from the liver type-PFK2 to the more active ubiquitous PFK2 isoenzyme, which responds to HIF-1α activation and increases fructose-2,6-bisphosphate concentration and glycolytic flux [61]. The opposite is true when macrophages are activated by interleukin (IL)-4 and IL-13, which promote the alternative phenotype, often termed M2 macrophages, which are associated with tissue repair and humoral immunity, anti-inflammatory cytokine production, reduced expression of MHC-II, and antigen presentation [61]. They exhibit enhanced OXPHOS and much lower rates of glycolysis, and have no detectable PFKFB3, expressing PFKFB1 instead. In human hepatocellular carcinoma, Chen et al. purified monocytes/macrophages from peripheral blood and found that PFKFB3 in TAMs not only modulated the cellular metabolic switch but also mediates the increased expression of PD-L1 by activating the nuclear factor-kappa B (NF-κB) signalling pathway in these cells [63]. The association between OXPHOS and the production of anti-inflammatory cytokines and glycolysis associated with the production of proinflammatory cytokines remains unclear. However, PPARγ co-activator-1β (PGC-1β) is a transcriptional co-activator that promotes oxidative metabolism, notably by upregulating the expression of genes involved in FAO in the M2 macrophage phenotype, which is a profound increase in the entire programme of fatty acid metabolism, including the uptake and oxidation of fatty acids and mitochondrial biogenesis, depending on the activation of signal transducer and activator of transcription 6 (STAT6) [64](Fig. 3). Notably, in the B16 melanoma tumour model, STAT6 has been demonstrated to induce M2 macrophage polarization and mediate the suppression of TRIM24 expression in M2 macrophages, contributing to the induction of an immunosuppressive tumour niche [65]. In addition, in the pentose phosphate pathway (PPP), the carbohydrate kinase-like (CARKL) protein, also known as sedoheptulose kinase (SHPK), plays a key role in regulating macrophage metabolism and can influence macrophage polarization, which catalyses the production of sedoheptulose-7-phosphate (S7P) as a rate-limiting step for balancing metabolic intermediates of non-oxidative PPP and glycolysis [66]. SHPK is downregulated upon LPS stimulation both in vitro and in vivo, and downregulation of SHPK is essential for M1-like metabolic reprogramming. Furthermore, SHPK antagonizes LPS-induced cytokine production (i.e., TNF-α and IL-6) by inhibiting NF-κB. Therefore, the negative effect of SHPK activity on M1 macrophage function raises the possibility that changes in glucose metabolism influence the inflammatory properties of M1 macrophages (Fig. 2). LPS-induced M1 macrophages also displayed increased glycolysis and decreased oxygen consumption (oxygen consumption rate, OCR) [67]. This contrasts sharply with IL-4-polarized M2-like macrophages, whose metabolic profile is similar to that of unpolarized macrophages. Two important metabolites generated by oxidative PPP are nicotinamide adenine dinucleotide phosphate (NADPH) and ribulose-5-phosphate (Ru5P) [68, 69]. If the cellular need for NADPH exceeds nucleotide biosynthesis, Ru5P passes into the non-oxidative arm of the PPP to generate F6P and G3P, which enter glycolysis again. M1 macrophages drive several processes that require a high amount of NADPH, notably NADPH oxidase-dependent respiratory burst and glutathione biosynthesis, to buffer reactive oxygen species (ROS) [70]. ROS can further activate NF-kB signalling to promote PD-L1 transcription and release of immunosuppressive chemokines from TAMs [71]. In triple-negative breast cancer models, ROS inducers such as paclitaxel, glutathione synthesis inhibitor, and buthionine sulphoximine can significantly promote ROS accumulation and elevate PD-L1 transcription both in vitro and in vivo experiments [71]. In contrast, ectopic expression of SHPK reduces the oxidative PPP flux and promotes an oxidative state (increased GSSG and NAD +) characteristic of M2-like polarization. Maintenance of a high NADH/NAD + ratio induced by LPS stimulation may enhance NF-κB binding activity and favour M1 macrophage differentiation [72]. In traumatic brain injury (TBI), delayed NADPH oxidase 2 (NOX2) activation induces NF-κB activation, amplifies neuroinflammation, enhances M1 polarization, and increases myeloid-mediated neurotoxicity [73]. Furthermore, NOX2-dependent ROS production occurs upstream of ATM activation, which is required for ionizing radiation-elicited macrophage activation and for macrophage reprogramming towards a proinflammatory phenotype after treatment with IFN-γ, LPS, or chemotherapeutic agents through the regulation of mRNA levels and post-translational modifications of IFN regulatory factor 5 (IRF5) [74]. A metabolic transition towards glycolysis, reminiscent of the Warburg effect, occurs in LPS-stimulated macrophages. Mitochondrial OXPHOS was reduced, but anaerobic glycolysis was enhanced. LPS elevates the transcription level of HIF-1α via the mitogen-activated protein kinase (MAPK) and NF-κB pathways, with a decrease in the mRNA levels of TLR4-dependent prolyl hydroxylase (PHD) mRNA levels. The LPS-induced decrease in OXPHOS leads to the accumulation of intermediate metabolites in the tricarboxylic acid cycle, especially succinic acid [75]. Succinic acid can be transferred from the mitochondria to the intracellular space, inhibit the activity of the PHD enzyme, and increase HIF-1α by promoting its stability. The depletion of HIF-1α in macrophages results in decreased production of IL-1β, which unsensitized mice to LPS-induced endotoxic shock with a lower mortality rate than wild-type mice.

Macrophages often accumulate in large numbers in areas of hypoxia, a prominent feature of various inflamed and diseased tissues, including malignant tumours, atherosclerotic plaques, myocardial infarcts, synovia of joints with rheumatoid arthritis, healing wounds, and sites of bacterial infection, where hypoxia affects the function of macrophages [76]. A recent study found that macrophages exert enhanced phagocytic clearance of apoptotic cells (efferocytosis) upon chronic physiological hypoxia [77]. In this process, macrophages flux glucose into PPP and promote NADPH production, which further induces phagolysosomal maturation and redox homeostasis to enhance efferocytosis. Thus, the macrophages play an important role in maintaining body homeostasis through efferocytosis under physiological hypoxic conditions. As an important molecule that regulates macrophage function under hypoxic conditions, HIF-1α can promote glycolysis and PPP by inducing the expression of related enzymes and transcriptional effectors, thereby affecting the biological functions of macrophages. For example, in hepatocellular carcinoma, the glycolytic enzyme pyruvate kinase M2 (PKM2), induced by hepatoma cell-derived fibronectin 1, can regulate macrophage glycolysis in a HIF-1α-dependent manner [78]. PKM2 is a protein kinase that regulated aerobic glycolysis [79, 80]. Follistatin-like protein 1 (FSTL1) promotes PKM2 phosphorylation and nuclear translocation via direct binding, induces PKM2-dependent glycolysis, and promotes M1 polarization [81]. Simultaneously, HIF-1α promotes the expression of proinflammatory genes in macrophages, enhances phagocytosis, affects the production of anti-microbial peptides and granzyme, and plays an important role in inflammatory response. Loss of HIF-1α reduces the bactericidal activity of macrophages and the secretion of proinflammatory cytokines. It has been found that HIF-1α can contribute to the synthesis of iNOS and to the other hypoxia response elements (HRE)-dependent transcriptional activity when stimulated synergistically by LPS or hypoxia [82]. Zhang and colleagues have demonstrated that M2 macrophages enhance 3-phosphoinositide-dependent protein kinase 1 (PDPK1)-mediated phosphoglycerate kinase 1 (PGK1) threonine (T) 243 phosphorylation in tumour cells by secreting IL-6 [83]. This phosphorylation facilitates a PGK1-catalysed reaction towards glycolysis by altering substrate affinity. In addition, PGK1 T243 phosphorylation correlates with PDPK1 activation, IL-6 expression, and macrophage infiltration in human glioblastoma (GBM) and correlates with the malignancy and prognosis of human GBM [83]. As has been descripted, hypoxia and inflammation are critical factors that influence the hepatocellular carcinoma microenvironment. TAMs secrete more IL-1β under moderate hypoxic conditions because of the increased stability of HIF-1α, which induces necrotic debris in hepatocellular carcinoma cells. Necrotic debris further induce IL-1β secretion via TLR4/TRIF/NF-κB signalling. However, overexpression of HIF-1α leads to epithelial–mesenchymal transition (EMT) and metastasis in hepatocellular carcinoma cells [84]. Furthermore, hepatocellular carcinoma-derived IL-8 promotes a pro-oncogenic inflammatory microenvironment by inducing M2-type TAMs and indirectly promoting EMT [85]. Additionally, hypoxic conditions can suppress forkhead box O1 (FoxO1) expression, which positively regulates MHC-II genes by binding to the promoter region of *Ciita*, the master activator of MHC-II genes. Yang et al. used FoxO1 conditional knockout mice to confirm that loss of FoxO1 in TAMs results in reduced MHC-II expression [86]. In the TME with high lactate content, prolonged lactic acidosis induces the differentiation of monocytes into macrophages with a phenotype that includes tumour-promoting and inflammatory characteristics (VEGF^hi^ CXCL8^+^IL-1β^+^). In vitro activation of macrophages at pH 6.8 in vitro enhanced the IL-4-driven phenotype and contributed to prostate carcinogenesis [87]. These effects of lactate require its metabolism and are associated with HIF-1α stabilization. The expression of lactate-induced genes is dependent on autocrine macrophage-CSF (M-CSF) consumption [88]. The tumour-derived soluble molecule succinate activates succinate receptor 1 (SUCNR1) signalling to polarize macrophages into TAMs and promote tumour cell migration and invasion as well as metastasis by the SUCNR1-triggered PI3K-HIF-1α axis [89]. High concentrations of lactate within the anaerobic tumour environment activate the mechanistic target of rapamycin complex 1 (mTORC1), which subsequently suppresses the transcription factor EB (TFEB)-mediated expression of the macrophage-specific vacuolar ATPase subunit ATP6V0d2, which targets HIF-2α but not HIF-1α, for lysosome-mediated degradation [90]. Hypoxia can also induce lactate production via glycolysis, which acts as a precursor for stimulating histone lactylation. Histone lactylation has different temporal dynamics from acetylation. In the late phase of M1 macrophage polarization, increased histone lactylation induces homeostatic genes involved in wound healing, including Arg1 [91]. Previous studies have confirmed that hypoxic TAMs play an important role in promoting tumour angiogenesis [24, 92]; however, whether metabolic changes can reverse this effect is ambiguous. Wenes et al. found elevated expression of REDD in hypoxic TAMs from Lewis lung carcinomas, orthotopic E0771 breast cancer, and spontaneous PyMT mammary tumours [93]. To further understand these mechanisms, they constructed chimeric mice and revealed that REDD1 can hinder the glycolysis of TAMs and enhance their proangiogenic function by inhibiting mTOR. Furthermore, REDD1-deficient TAMs compete with tumour endothelial cells to utilize glucose, which stabilizes endothelial cells and blocks abnormal blood vessel formation. Therefore, TAM metabolism also plays regulatory roles in tumour angiogenesis.

### Lipid metabolism

Lipidomic studies have confirmed that lipid metabolism is related to macrophage activation [94, 95]. However, when excess cholesterol is absorbed, abnormal cholesterol metabolism in macrophages leads to several pathological changes. Using index and transcriptional single-cell sorting, researchers have revealed a novel lipid-associated macrophage subset, which is characterized by lipid receptor Trem2 expression in both mice and humans during obesity [96]. Mechanistically, they used Trem2-deficient mice to confirm that Trem2 plays an important role in preventing adipocyte hypertrophy and in regulating systemic cholesterol levels. Thus, lipid-associated macrophages may be effective targets in for metabolic diseases. Abundant endoplasmic reticulum and free cholesterol in macrophages promotes the esterification of cholesterol acyltransferase 1 (ACAT1), which in turn leads to the production of more free cholesterol and increases the inflammatory signals induced by lipid rafts, especially TLRs and NF-κB. This signalling pathway causes changes in the lipid metabolism of macrophages. TLR agonists promote the biosynthesis of atypical arachidonic acid from eicosapentaenoic acid (EPA) and docosahexaenoic acid (DHA) and produce anti-inflammatory lipid regulators such as resolvin and protectin during tissue repair by M2 macrophages. Notably, Endogenous oxidized lipids can simultaneously promote OXPHOS and aerobic glycolysis in LPS-stimulated phagocytes [97]. Studies have also demonstrated that fatty acid absorption and oxidation were significantly increased in IL-4 stimulated M2 macrophages and inhibited in M1 macrophages [98, 99] (Fig. 2). The polarization of human macrophages is related to the levels of glycosphingolipid regulators, sphingosine and ceramide. The arachidonic acid pathway can describe the biosynthesis of proinflammatory lipid mediators, such as prostaglandin E2 (PGE2) and PGD2, in the inflammatory response, which is one of the ligands of the liver X receptor (LXR) in the nucleus. LXR prevents arachidonic acid from remodelling TLR4 response elements, thereby inhibiting TLR4-activated macrophages. Therefore, the anti-inflammatory effects of arachidonic acid are partly dependent on the LXR pathway. However, there are significant differences in the expression of arachidonic acid pathway-related enzymes between M1 and M2 macrophages in humans, which express high level of cyclooxygenase 2 (COX2) and low levels of COX1, leukotriene a4 hydrolase (LTA4H), and arachidonate 5-lipoxygenase (ALOX5) upon stimulation with IFN-γ and/or LPS, whereas ALOX15 and COX1 increased markedly after treatment of macrophages with IL-4. The synthesis of arachidonic acid is catalysed by 24-dehydrocholesterol reductase (DHCR24), which inhibits the expression of DHCR24 in mice fed a high-fat diet, leading to M1-type activation of macrophages. 5-Lipoxygenase (5-LO) is key to the synthesis of leukotrienes, which are potent proinflammatory lipid mediators involved in chronic inflammatory diseases, including cancer. The expression and activity of 5-LO in TAMs were reduced upon co-culture with dying cancer cells through Mer tyrosine kinase (MerTK)-dependent recognition of apoptotic cancer cells, which can be repressed by the proto-oncogene c-Myb at the transcriptional level [100]. Notably, blockade of MerTK resulted in the accumulation of apoptotic cells within tumours and triggered a type I interferon response. Treatment of tumour-bearing mice with the anti-MerTK antibody stimulated T cell activation and synergized with anti-PD-1 or anti-PD-L1 therapy. Mechanistically, extracellular ATP acts via P2X7R to enhance the transport of extracellular cyclic guanosine monophosphate–adenosine monophosphate (cGAMP) into macrophages and subsequent stimulator of interferon gene (STING) activation [101]. Treg cells promote M2-like TAMs by repressing the CD8^+^ T cell-IFN-γ axis by blocking the activation of sterol regulatory element-binding protein 1 (SREBP1)-mediated fatty acid synthesis [102]. Cytosine–guanine dinucleotide (CpG) activation engenders a metabolic state that requires fatty acid oxidation (FAO) and the shunting of tricarboxylic acid (TCA) cycle intermediates for de novo lipid biosynthesis. This integration of metabolic inputs is underpinned by carnitine palmitoyl transferase 1A and adenosine tri-phosphate citrate lyase, which together impart macrophages with anti-tumour potential capable of overcoming inhibitory CD47 on cancer cells [103]. Macrophages from both human and murine tumour tissues are enriched with lipids owing to increased lipid uptake. TAMs express elevated levels of the scavenger receptor CD36, accumulate lipids, and use FAO instead of glycolysis for energy [104]. High levels of FAO promote mitochondrial OXPHOS, ROS, Janus kinase 1 (JAK1) phosphorylation, and Src homology 2 domain-containing protein tyrosine phosphatase 1 (SHP1) dephosphorylation, leading to STAT6 activation and transcription of genes that regulate TAM generation and function [105]. Peroxisome proliferator-activated receptor (PPAR) is a ligand-dependent transcription factor that acts as a fatty acid receptor to regulate glucose and lipid metabolism, and can be further divided into three subgroups: PPARα, PPARδ, and PPARγ [106]. PPARα and PPARγ are widely expressed in human and mouse monocytes and macrophages and inhibit the expression of proinflammatory genes in macrophages. Therefore, PPAR is generally believed to prevent M1 polarization, and its effect on M2 polarization has gradually been discovered in recent years. PPARγ controls the expression of genes encoding molecules that mediate various aspects of lipid metabolism, including storage, lipolysis, and cholesterol efflux [107]. PPARγ, which affects M2 polarization, mainly promotes fatty acid omega-oxidation and mitochondrial generation at the transcriptional level, and can couple with PGC-1β to directly regulate the production of Arg1, a hallmark of M2 macrophages. Similarly, knockout of PPARγ in macrophages both in vivo and in vitro inhibited the activation of M2 macrophages, and decreased Arg1 production [108, 109]. Mice fed a high-fat diet that specifically knocked out PPARγ in myeloid cells were more prone to obesity and insulin resistance owing to damage to mitochondrial function. Caspase-1 promotes TAM differentiation by cleaving PPARγ at Asp64, which translocates into the mitochondria where it directly interacts with medium-chain acyl-CoA dehydrogenase (MCAD). This binding event attenuates MCAD activity and inhibits fatty acid oxidation, thereby leading to the accumulation of lipid droplets and promotion of TAM differentiation [110]. IL-4 can also induce PPARδ transcription and act synergistically with STAT6 to promote M2 activation. Unlike PPARγ, PPARδ is not required for the oxidative metabolism. It is worth mentioning that the co-regulation of STAT6 and PPARγ affects the transcription of genes related to fatty acid oxidation, leading to the polarization of macrophages into M2, and the metabolic level shifts from glycolysis to fatty acid oxidation. Notably, rosiglitazone, a PPARγ agonist, can partially decrease C–C motif chemokine ligand 2 (CCL2) secretion by tumour cells and reduce the infiltration of TAMs to the irradiated tumour site, thereby delaying tumour regrowth after radiotherapy, suggesting that the combination of the PPARγ agonist rosiglitazone with radiotherapy can enhance the effectiveness of radiotherapy [111]. PGC-1β, a transcriptional co-stimulator of PPAR, increases the expression of genes related to fatty acid oxidation and promotes OXPHOS. During M2 polarization by IL-4, fatty acids metabolism was significantly improved, as well as the oxidation and absorption of fatty acid and the number of mitochondria, mainly because IL-4 activates the transcription factor STAT6 and further induces the production of PGC-1β. Intracellular overexpression of PGC-1β promotes M2 polarization and alleviate macrophage-related inflammatory responses. In contrast, conditional knockout of PGC-1β inhibited intracellular OXPHOS and M2 function, significantly promoting the M1 inflammatory response activated by LPS. Similarly, prostaglandin E2 (PGE2) can elevated mitochondrial OXPHOS by inhibiting PPARγ and inducing alterative macrophage polarization [112]. Monoacylglycerol lipase (MGLL) can lead to lipid overload in TAMs, which functionally inhibits CB-2 cannabinoid receptor-dependent tumour progression in inoculated and genetic cancer models. Mechanistically, MGLL deficiency promotes CB-2/TLR4-dependent macrophage activation, which further suppresses the function of tumour-associated CD8^+^ T cells [113]. Ovarian cancer cells promote membrane cholesterol efflux and lipid raft depletion in macrophages. Increased cholesterol efflux promotes IL-4-mediated reprogramming, including the inhibition of IFN-γ-induced gene expression, which reverts the tumour-promoting functions of TAMs and reduces tumour progression [107, 114].

### Amino acid metabolism

In macrophages, intracellular metabolism of L-Arg is mainly regulated by two enzymes: iNOS and Arg1. iNOS catalyses the conversion of L-Arg into NO and L-citrulline. NO plays a bactericidal role, and L-citrulline is utilized in the urea cycle. Arg1 catalyses L-Arg to produce ornithine and uridine, and promote the formation of polyamines in collagen synthesis, cell proliferation, and tissue remodelling. Arg1 activity can be significantly elevated in M2 macrophage upon stimulation with IL-4 in mice, but similar results have not been found in human macrophages. Elevated amino acid catabolism is common in several cancers. Glioblastoma can produce large amounts of branched-chain ketoacids (BCKAs), which can be taken up and reaminated into branched-chain amino acids (BCAAs) by TAMs. Exposure to BCKAs can reduce the phagocytic activity of macrophages [115]. SLC7A5, an important transporter, has been demonstrated to mediate the uptake of amino acids in tumours and T cells, and it has been confirmed that SLC7A5-mediated metabolic reprogramming plays a major role in macrophages polarization [116]. SLC7A5 promotes the release of proinflammatory cytokines from macrophages by inducing leucine influx and upregulating glycolytic reprogramming via the mTORC1 signalling pathway. Ornithine decarboxylase (ODC) is the rate-limiting enzyme in polyamine biosynthesis and restricts M1 macrophage activation in gastrointestinal (GI) infections, which augments epithelial injury-associated colitis and colitis-associated carcinogenesis (CAC) by impairing M1 responses that stimulate epithelial repair, anti-microbial defence, and anti-tumour immunity [117]. Arginase 2 (ARG2) drives neuroblastoma cell proliferation via regulation of arginine metabolism, which polarizes infiltrating monocytes to an M1 macrophage phenotype, releasing IL-1β and TNF-α in an RAC-α serine/threonine protein kinase (AKT)-dependent manner [118]. Serine is a substrate for nucleotide, NADPH, and glutathione (GSH) synthesis. In macrophages, serine is required for optimal LPS induction of IL-1β mRNA expression, but not for inflammasome activation [119].

## Metabolites that regulate macrophage polarization

Changes in macrophage metabolism are accompanied by intermediate metabolic alterations. Specifically, the metabolites produced during in TCA cycle play important roles in the modulation of macrophages (Table 3).

**Table 3:** Potential druggable targets in reprogramming metabolism

Drug	Target	Effect	Function	Refs.
Depletion of Zeb1	Zeb1	Weakened aerobic glycolysis	Reprogrammed TAM polarization	[150]
SGLT1 inhibitor	SGLT1	Decreased glycolysis	Inhibited M2 polarization	[152]
MIF-CD74 blockade	MIF-CD74	Decreased lactate production	Promoted M1 infiltration	[156]
TLR9 agonist CpG ODN	Wnt2b/β-catenin	Decreased glycolysis	Suppressed M2 polarization	[159]
Rapamycin	mTOR	Remodelled glycolysis metabolism	Reprogrammed M2 to M1	[161]
GARP or integrin inhibitors	GARP/integrin	Upregulated glucose metabolism and OXPHOS gene expression	Restored M1 anti-tumour effect	[163]
Nanoplatform deliver MGLL siRNA and CB-2 siRNA	MGLL/CB-2	Inhibited free fatty acid production	Reprogrammed TAMs to polarize into M1 macrophages	[164]
ABHD5 inhibitor	ABHD5/SRM	Inhibited lipolysis of triglycerides into diglycerides and free fatty acids	Reprogrammed TAM polarization	[165, 166]
PERK inhibitor	PERK	Inhibited glutamine utilization and α-KG concentration	Reduced TAM activity	[167]
Slit2 activator	Slit2	Increased glycolysis, reduced FAO, reduced α-KG-to-succinic acid ratio	Promoted M1 polarization	[168,169]
Nanotherapeutics loaded with TLR7/8 agonist and FAO inhibitor	TLR7/8	Inhibited TCA cycle, upregulated glycolytic metabolic pathway	Reprogrammed M2 to M1	[35]
RIPK3 upregulation	RIPK3	Increased anaerobic glycolysis	Contributed to M1 polarization	[171,172]

### Succinate

Succinate is an intermediate product of the TCA cycle [120], and it is significantly increased in response to LPS stimulation [75]. LPS increased levels of succinate played crucial roles in stabilizing HIF-1α and promoting IL-1β production in proinflammatory macrophages by impairing PHD activity. This finding suggests that succinate directly regulates the HIF-1α pathway, thereby influencing macrophage function. Furthermore, in vitro and in vivo studies have shown that tumour-derived succinate in the TME can activate SUCNR1, leading to the polarization of macrophages into TAMs. Additionally, succinate promotes tumour cell migration and invasion. High levels of succinate and SUCNR1 expression are associated with poor clinical outcome in lung cancer [89].

### Itaconate

Itaconate has been shown to be produced by macrophages after LPS stimulation [121]. LPS-treated macrophages exhibit high expression of immune-responsive gene 1 (IRG1), which catalyses the decarboxylation of cis-aconitate to produce itaconate [122]. Silencing the IRG1 gene in macrophages significantly reduces itaconic acid production during bacterial infections, highlighting the important role of IRG1 in regulating immune defence and itaconate production. However, Lampropoulou et al. demonstrated that itaconate exerted anti-inflammatory effects by inhibiting succinate dehydrogenase (SDH) production, leading to increased succinate accumulation and decreased levels of mitochondrial reactive oxygen species (ROS), which subsequently inhibited the release of proinflammatory cytokines [123]. Additionally, itaconate and its derivative 4-octyl itaconate (OI) inhibited NLRP3 inflammasome activation, thereby limiting inflammation in a urate-induced peritonitis model [124]. Similarly, Hoyle et al. found that OI the derivative 4OI and dimethyl fumarate (DMF) effectively inhibited the production of proinflammatory cytokines in murine BMDMs, mixed glia and organotypic sliced hippocamp cultures in response to LPS [125]. Moreover, itaconate alkylated cysteine residues on the protein Kelch-like ECH-associated protein 1 (KEAP1) in both mouse and human macrophages, leading to the negative modulation of the expression of nuclear factor 2 (Nrf2) [126, 127], a transcription factor essential for activating antioxidant and anti-inflammatory responses. Thus, itaconate may exert anti-inflammatory effects in a Nrf2-dependent manner. Itaconate and its derivative dimethyl itaconate (DI) also exerted anti-inflammatory effects through an Nrf2-independent pathway [128]. They induce electrophilic stress to inhibit the IκBζ-ATF3 inflammatory axis. Additionally, the itaconate derivative OI has been found to decrease the activity of the glycolytic enzyme GAPDH, thereby blocking glycolytic flux, reducing aerobic glycolysis, and preventing proinflammatory TAM activation [129]. Furthermore, itaconate suppressed M2 macrophage polarization [130], as it inhibited JAK1 and STAT6 activation.

Researchers have discovered that itaconate promotes tumour growth by mediating cross talk between macrophages and cancer cells in peritoneal tumours [131]. Itaconate enhanced OXPHOS-driven ROS expression and induced MAPK activation, mediated through ROS signalling in tumour cells. Blocking itaconate and IRG1 significantly inhibited tumour progression and may thus be an effective therapeutic strategy. In colorectal cancer, itaconate downregulated PPARγ expression and increased the secretion of anti-inflammatory cytokines by M2 macrophages, thereby promoting tumourigenesis [132]. A recent study demonstrated that itaconate suppressed CD8^+^ T cell proliferation, and blocking itaconate restored anti-tumour immunity in mouse models of melanoma [133]. Moreover, itaconate inhibitors synergized with immune checkpoint inhibitors, resulting in a greater anti-tumour effect on melanoma.

### α-Ketoglutarate

α-Ketoglutarate (α-KG) is generated in various metabolic pathways. Previous studies have shown that α-KG is produced through the oxidative decarboxylation of isocitrate by isocitrate dehydrogenases (IDHs) [134]. A recent study found that glutamine deprivation in mouse BMDMs inhibited the expression of M2-like marker genes but promoted the expression of M1-like marker genes after LPS stimulation. However, the precise mechanisms by which glutamine metabolism regulates macrophage polarization remain unclear. Liu et al. discovered that α-KG, generated from glutamine, promoted M2 polarization through the Jmjd3 signalling pathway [47]. Moreover, α-KG inhibited M1 macrophage function by suppressing the NF-κB pathway in a PHD-dependent manner. α-KG effectively activated the PHD enzyme, leading to a significant reduction in IKKβ activation, which is necessary for NF-κB pathway activation. As mentioned earlier, succinate stabilizes HIF-1α, whereas α-KG destabilizes it [47, 75]. Therefore, an elevated α-KG/succinate ratio may promote M2 macrophage polarization, while a reduced α-KG/succinate ratio may facilitate M1 macrophage reprogramming by targeting HIF-1α-mediated aerobic glycolysis. In mouse BMDMs, type I interferon (IFNβ) increased IRG1 expression, promoting the production of itaconate and succinate while inhibiting α-KG production [135]. Downregulation of the α-KG/succinate ratio suppressed M2 macrophage polarization through the JMJD3/IRF4-mediated pathway. Thus, IFNβ plays a significant role in regulating macrophage polarization by controlling the α-KG/succinate ratio. Although the involvement of glutamine-derived α-KG in regulating M2 macrophage activation has been established, the specific mechanisms remain unclear. Zhou et al. revealed that after IL-4 stimulation, glutaminolysis promoted α-KG accumulation and reprogrammed M2 polarization through the SENP1-Sirt3 axis in BMDMs [136]. The SENP1-Sirt3 axis deacetylated glutamate dehydrogenase 1 (GLUD1), an acetylated protein in the mitochondria, and activated GLUD1 induced α-KG accumulation, promoting M2 macrophage polarization [136].

### Citrate

Citrate has been shown to induce pro- or anti-inflammatory macrophage polarization through different mechanisms [137]. Previous studies have demonstrated that citrate derived from mitochondria promoted the activation of proinflammatory macrophages. The mitochondrial citrate carrier (CIC) facilitated the export of citrate from mitochondria in LPS-activated macrophages, leading to increased HIF-1α expression. HIF-1α, in turn, upregulated IRG1, resulting in itaconate production [138]. Inhibition of CIC suppressed citrate accumulation and enhanced mitochondrial oxidation by blocking the itaconate shunt, ultimately causing a switch from M1 to M2 BMDM polarization after LPS stimulation. Moreover, in human macrophage cells derived from histiocytoma, the proinflammatory cytokines TNF-α and IFN-γ were required for mitochondrial CICs production of nitric oxide and prostaglandin [139].

In contrast, Covarrubias et al. discovered that IL-4 activates the Akt and mTORC1 signalling pathways. Activation of Akt-mTORC1 pathway promoted the conversion of citrate into acetyl-CoA through the activation of ATP-citrate lyase (ACLY). This process increased histone acetylation and promoted the expression of M2 genes, ultimately leading to M2 macrophage activation [140].

## Metabolite-regulated macrophage polarization influences cancer outcome

As a major component of immune cells in the TME, TAMs play pivotal roles in tumour progression. Strategies targeting TAMs focus mainly on TAM deletion, inhibition of TAM recruitment, and reprogramming of TAM polarization [15, 141, 142]. However, the therapeutic effects of these approaches are still not ideal, and there is an urgent need for new effective therapies targeting TAMs in tumour treatment. Recent studies have highlighted a role for metabolic reprogramming in controlling macrophage function and polarization, leading to various clinical experiments aimed at regulating macrophages (as summarized in Table 4). Because a limited number of reviews are available on this topic, we summarize the metabolic reprogramming of macrophages during cancer treatment in Table 5.

**Table 4:** Characteristics of the different macrophage subtypes

Phenotype	Stimuli	Markers (human)	Markers (mouse)	Functions
M1	IFN-γ, TNFα, IL-1β, LPS	CD11b, CD11c, CD80, HLA-DR, IL-1β, IL-8, TNFα, IL-12	CD11b, F4/80, CD80, CD86, Ly6c, MHC-II, iNOS, IL-1β, IL-8, TNF-α, IL-12	Proinflammatory, anti-tumour
M2a	IL-4, IL-13	CD206, CD163, IL-10, TGF-β, CCL17, CCL18, CCL22, CCL24	CD163, Arg1, IL-10, TGF-β, CCL17, CCL18, CCL22, CCL24	Anti-inflammatory
M2b	IL-1β, LPS	CD86, IL-10, IL-12, IL-6, TNF-α	IL-10, IL-12, IL-6, TNF-α	Immunoregulation, tumour progression
M2c	IL-10, TGF-β, glucocorticoids	CD163, CD206, IL-10, TGF-β,	Arg1, IL-10, TGF-β,	Angiogenesis, phagocytosis, wound healing
M2d	TLR ligand, LPS, IL-6	IL-10, VEGF	IL-10, VEGF	Tumour progression, immunosuppressive, angiogenesis

**Table 5:** Targeting cell metabolism for cancer treatment

Targets	Drugs	Clinical phase	Conditions	Sponsor	Gov identifier
Tyrosine kinase	PLX3397	1	Prostate adenocarcinoma	Barbara Ann Karmanos Cancer Institute	NCT02472275
Cholesterol	Tesco	Not applicable	Breast cancer	University of Leeds	NCT04147767
mTOR	Sirolimus	1/2	Pancreatic cancer	Second Affiliated Hospital, School of Medicine, Zhejiang University	NCT03662412
Glucose metabolism	Metformin	2	Breast cancer	Oxford University Hospitals NHS Trust	NCT01266486
		1	Head and neck squamous cell cancer	Sidney Kimmel Cancer Center at Thomas Jefferson University	NCT02083692
		1	Cancer of head and neck	West Virginia University	NCT02402348
		2	Lung cancer	M.D. Anderson Cancer Center	NCT02285855
		1	Endometrial cancer	M.D. Anderson Cancer Center	NCT01205672
	2-DG	1/2	Prostate cancer	Rutgers, The State University of New Jersey	NCT00633087
			Intracranial neoplasms		NCT00247403
			Neoplasm metastasis		
PPARγ	Rosiglitazone	2	Melanoma	Dan Zandberg	NCT04114136
			Nsclc		
			Hepatocellular carcinoma		
	Dichloroacetate	1	Head and neck cancer	Daniel T. Chang	NCT01163487
	Pioglitazone	2	Cancer of the pancreas	University of Texas Southwestern Medical Center	NCT01838317
Cholesterol	Evolocumab	1	Pancreatic ductal adenocarcinoma	CHU de Quebec-Universite Laval	NCT04862260
		1	Glioblastoma	Duke University	NCT04937413
	Atorvastatin		Pancreatic cancer		
	Ezetimibe		Pancreas cancer		
			Metastatic cancer		
HMG-CoA reductase	Rosuvastatin	4	Prostate cancer metastatic	National Cancer Institute, Egypt	NCT04776889
Arginase	INCB001158	1/2	Metastatic cancer	Incyte Corporation	NCT02903914
			Solid tumours		
			Colorectal cancer		
			Gastric cancer		
		1	Advanced solid tumours	Advanced solid tumours	NCT03910530
			Advanced solid tumours		
		1/2	Biliary tract cancer	Incyte Corporation	NCT03314935
			Colorectal cancer		
			Endometrial cancer		
		1/2	Solid tumours	Incyte Corporation	NCT03361228

### Regulating TAM function through aerobic glycolysis

Tumour metastasis is a leading cause of treatment failure and recurrence. Several cancer-related features contributes to the development of the pre-metastatic niche, including inflammation [143], angiogenesis [144], immunosuppression [145], and reprogramming [146]. Among the various immune cells in the pre-metastatic niche, macrophages have gained significant attention [147, 148]. Targeting macrophages shows potential for suppressing tumour metastasis. However, the precise mechanisms underlying the regulation of macrophage polarization and function in the pre-metastatic niche have remained unclear. Morrissey and colleagues have revealed that tumour-derived exosomes polarized macrophages into an immunosuppressive subtype via metabolic reprogramming, which increased glucose uptake through the NF-κB pathway, elevated NOS2 expression, and inhibited mitochondrial OXPHOS, which favoured the conversion of pyruvate into lactate in the lung cancer context. These outcomes were confirmed both in vitro in F4/80^+^ peritoneal macrophage experiments and in vivo in animal experiments [149]. Notably, increased lactate levels established a feedback metabolism to the NF-κB pathway and elevated PD-L1 expression in macrophages. Ultimately, this resulted in tumour progression and metastasis. Therefore, reprogramming macrophage metabolism by regulating tumour-derived exosomes might be an effective anti-tumour therapeutic strategy.

Zinc finger E-box binding homeobox 1 (Zeb1), a transcription factor, has been demonstrated to reprogramme TAMs to become immunosuppressive M2-like TAMs in human breast cancer samples. Zeb1 induced aerobic glycolysis in TAMs, leading to increased lactate production, which forms an acidic environment that promotes tumour progression and metastasis [150]. In a hypoxic environment, Zeb1 promoted the expression of glycolytic-related enzymes through the PI3K/AKT signalling pathway. Depletion of Zeb1 inhibited PI3K/AKT activity and aerobic glycolysis, which may indicate that Zeb1 depletion is potential therapeutic strategy for breast cancer because it led to attenuate aerobic glycolysis and reprogrammed TAM polarization. Endocrine therapy has led to major advances in oestrogen receptor (ER)-positive breast cancer treatment. However, resistance to endocrine therapy remains a challenge. TAMs play important roles in inducing endocrine therapy resistance, but the specific mechanism underlying its effect remains unclear [151]. Niu et al. discovered that overexpression of sodium/glucose co-transporter 1 (SGLT1) enhanced glycolysis in ER-positive breast cancer cells and promoted M2-like TAM polarization mediated through the HIF1α pathway. In turn, M2-like TAMs upregulated SGLT1 expression via EGFR/PI3K/Akt signalling, leading to endocrine therapy resistance in ER-positive breast cancer cells [152]. Therefore, targeting SGLT1 may be an effective treatment for overcoming endocrine therapy resistance in breast cancer and reprogramming TAM polarization. Triple-negative breast cancer, a distinct variant of breast cancer with a unique pathology, shows poor responses to immunotherapy because of the high lactic acid metabolism rate and high antioxidant levels in the TME [153]. New nanodrugs have been developed to polarize TAMs into the anti-tumour TAMs by reprogramming TAM metabolism, thereby enhancing the anti-tumour effects of TAMs [154, 155]. Macrophage migration inhibitory factor (MIF) is a well-characterized immunosuppressive factor that is secreted by immune cells and plays an important role in tumour immune escape by binding to its receptor CD74 [156, 157]. Previous studies have shown that MIF-CD74 inhibitors restored the anti-tumour immune function of macrophages and dendritic cells in metastatic melanoma [156]. Recently, Azevedo et al. revealed that blockade of the MIF-CD74 signalling pathway reprogrammed the metabolic pathway by decreasing lactate production and promoting M1-like macrophage conversion in the TME. Moreover, MIF-CD74 blockade combined with anti-CTLA-4 therapy elevated CD8^+^ T cell infiltration and inhibited melanoma progression and metastasis [158]. Hepatocellular carcinoma-derived polarization-promoting factors promoted TAM polarization to the M2-like phenotype by activating the Wnt2b/β-catenin/c-Myc signalling pathway, which enhanced TAM glycolysis. This effect was blocked by the TLR9 agonist CpG ODN, which inhibited Wnt2b/β-catenin pathway activation and suppressed the M2 polarization of TAMs in hepatocellular carcinoma samples, ultimately reversing the tumour-promoting effects of the TAMs both in vitro and in vivo [159].

Previous studies have verified a relationship between mTOR signalling and TAM repolarization [160], and a combination of mTOR inhibitors and anti-angiogenic therapy has achieved good results in clinical experiments [161]. However, the specific molecular mechanisms have not yet been elucidated. Chen et al. designed a liposomal system including the mTOR inhibitor rapamycin and the anti-angiogenic drug regorafenib, and found that this liposome effectively reprogrammed M2-like TAMs to M1-like TAMs by remodelling glycolytic metabolism and reducing lactic acid production via the mTOR pathway. This effect was confirmed with both CT26 colon cancer cells and a colorectal tumour model [162]. Therefore, anti-angiogenesis and mTOR inhibition may co-regulate the repolarization.

In addition to being mediated by cellular factors, metabolic reprogramming is also activated by direct cell–cell contact in pancreatic ductal adenocarcinoma (PDA). Zhang and colleagues used a PDA macrophage co-culture system, that is, an “orthotopic” PDA syngeneic mouse model, and human PDA specimens to confirm that PDA tumour cells promoted the reprogramming of the M1-like cell phenotype into the M2-like cell phenotype through direct interaction with M1-like macrophage but not M2-like macrophages, a process that was mediated by GARP and integrin αV/β8, inducing DNA methylation and downregulating glucose metabolism and OXPHOS gene expression [163]. Inhibition of GARP or integrin reversed this outcome and restored the anti-tumour effect of the M1-like macrophages.

### Regulating TAM function through lipid metabolism

Recently, researchers have found that abnormal lipid metabolism and TAMs lead poor prognosis in pancreatic cancer. Cao et al. found that MGLL was highly expressed in pancreatic cancer, while the function endocannabinoid receptor-2 (CB-2), which can regulate macrophage polarization, was also dysregulated in TAMs [164]. The group synthesized a nanoplatform that simultaneously delivered MGLL siRNA and CB-2 siRNA to inhibit free fatty acid production in the TME and reprogramme TAMs to polarize into a tumour-inhibiting M1-like TAMs. Tumour cells have been reported to promote cholesterol efflux and reduce lipid rafts formation in macrophages [107]. Compared to naïve macrophages, TAMs are associated with increased expression of genes related to cholesterol metabolism and cholesterol efflux. Cholesterol depletion in macrophages induces IL-4-mediated macrophage activation and polarization through the STAT6-PI3K pathway, and these IL-4-mediated macrophages exerted immunosuppressive functions and promoted tumour progression [107]. Therefore, the cholesterol metabolism pathway is likely a novel target for reprogramming TAM polarization and function. AB-hydrolase containing 5 (ABHD5) functions as a co-activator of adipose triglyceride lipase and plays a critical role in the lipolysis of triglycerides into diglycerides and free fatty acids [165]. Miao et al. found that ABHD5 is highly expressed in colorectal cancer-related TAMs, which inhibited spermidine synthase (SRM)-dependent spermidine production by suppressing C/EBPε expression and counteracting the anti-tumour effect of TAM-derived spermidine on colorectal cancer [166]. Therefore, the ABHD5/SRM/spermidine metabolic pathway is a novel therapeutic strategy for colorectal cancer treatment.

### Regulating TAM function through TCA cycle metabolism

RNA-sequencing (RNA-seq) analysis revealed that the gene expression of protein kinase RNA-like endoplasmic reticulum kinase (PERK) favours the polarization of M2 macrophages and is associated with macrophage metabolism, including glutamine metabolism, amino acid synthesis, lipid metabolism, and OXPHOS [167]. Raines and colleagues discovered that PERK mediated mitochondrial respiration and FAO to meet M2 macrophage energy demands. PERK stimulated α-KG production in M2 macrophages by activating phosphoserine aminotransferase 1 (PSTA1), which was necessary for M2 macrophage metabolic reprogramming, and supported JMJD3-mediated histone demethylation to promote immunosuppressive gene expression in macrophages. Furthermore, inhibition of the PERK signalling pathway inhibited glutamine utilization and α-KG concentration in M2 macrophages, reduced immunosuppressive TAM activity, and suppressed tumour progression. Therefore, the PERK signalling pathway may be an effective target for the treatment of cancers by reprogramming macrophage metabolism.

Slit2, a secretory glycoprotein, has been found to inhibit breast cancer progression [168]; however, the specific mechanism remains unknown. Kaul and colleagues used a spontaneous mammary tumour virus promoter–polyoma middle T antigen (PyMT) breast cancer mouse model and found that Slit2 promoted BMDMs polarization towards an anti-tumour phenotype and enhanced the anti-tumour immune response by increasing glycolysis and reducing FAO in BMDMs via the mTOR signalling pathway. Moreover, Slit2 treatment reduced the α-KG-to-succinic acid ratio and changed mitochondrial respiration metabolites in macrophage-derived from healthy human blood that had been treated with breast cancer patient plasma [169]. These finding suggest that, Slit2 may be an important therapeutic target for breast cancer because it reprogrammes macrophage metabolism.

Recently, new metabolic supramolecular nanotherapeutics loaded with a TLR7/8 agonist and an FAO inhibitor were synthesized, and they effectively inhibited the TCA cycle and upregulated the glycolytic metabolic pathway of TAMs in breast cancer. Ultimately, M2-like TAMs are reprogrammed to be M1-like TAMs, significantly reduced the tumour progression and metastasis rate [170]. RIPK3 has been demonstrated to play an important role in activating the pyruvate dehydrogenase complex E3 subunit and increasing anaerobic glycolysis [171]. Considering that M1 macrophage polarization is associated with aerobic glycolysis, RIPK3 may contribute to M1 polarization of proinflammatory macrophages. Researchers have found that RIPK3 is downregulated in hepatocellular carcinoma cells and can induce M2-like TAM polarization and recruitment by activating the PPAR pathway to reprogramme fatty acid metabolism [172]. In addition, the upregulation of RIPK3 or ablation of FAO switched TAMs from the M2-like to M1-like phenotype and may be a potential method of tumour immunotherapy and metabolism-targeted therapy.

### Regulating TAM function through amino acid metabolism

The metabolism of L-arginine changes during macrophage polarization, and two of the three L-arginine catalytic enzymes, iNOS and arginase 1, have been well studied. However, the third metabolic product of L-arginine creatine which functions in the immune system, remains unclear. Creatine uptake, which is mediated by Slc6a8, reprogrammed macrophage polarization by inhibiting the IFN-γ-JAK-STAT1 signalling pathway and suppressing the expression of the immune effector molecule, IFN-γ. Additionally, it led to upregulated IL-4-STAT6 pathway activation and promoted of immune suppressor production [173]. Therefore, creatine metabolism plays a key role in macrophage polarization and the immune response and may emerge as an important therapeutic target for treatments mediated via macrophage repolarization.

Methionine is an essential amino acid, and researchers have revealed that methionine and methionine adenosylmethionine (MAT) enzymes play significant roles in tumourigenesis and tumour progression [174, 175]. However, whether methionine and MAT enzymes are associated with macrophage polarization remains unclear. Zhang at el. found that the expression of the MAT enzyme MAT2A was significantly upregulated in CD14^+^ monocytes purified from gastric cancer patient’s tumour tissues, and methionine metabolism promoted M2 macrophage polarization through MAT2A action, while MAT2A induced the epigenetic activation of RIP1 expression. Inhibition of MAT2A hindered M2 macrophage polarization [176]. Hence, targeting the MAT2A-RIP1 pathway may be a meaningful therapeutic strategy to reprogramme TAM metabolism and induce TAM polarization.

### Regulating TAM function through phosphoinositide metabolism

Phosphoinositides constitute a very small percentage of membrane phospholipids and play important roles in signalling modulation [177]. PIP2 and PIP3 have been shown to regulate signal transduction through the PI3K/Akt signalling pathway [178]. Tumour necrosis factor α-induced protein 8-like 1 (TIPE1) has been demonstrated to be highly expressed in isolated peritoneal macrophages, BMDMs and cultured THP1 cells, in which it promoted M2 macrophage polarization by directly binding to PIP2 and PIP3, regulating their metabolic pathways [179]. In vitro and in vivo, TIPE1 blockade in macrophages inhibits PI3K/Akt pathway activity and abrogated the progression and metastasis of melanoma and liver cancer cells. Therefore, phosphoinositide signalling and metabolism may be effectively changed through TAM reprogramming and acquisition of an anti-tumour phenotype.

## Perspective

Recently, dramatic advances have been made in tumour immunotherapy. For example, immune checkpoint blockade therapy has been successful in reducing many types of solid tumour [180–182], and chimeric antigen receptor (CAR)-T therapy has also shown promising effects in the treatment of haematologic malignancies [183, 184]. However, the wide application of CAR-T cell therapy is limited due to severe toxicity, such as cytokine release syndrome (CRS) mediated by cytokines derived from macrophages [185]. Therefore, targeting TAMs is a necessary and promising strategy for tumour immunotherapy. TAM infiltration has been associated with poor prognosis in many malignant tumours [30, 186], but little is known about the effects of TAM metabolic changes on tumour progression. Metabolic reprogramming leads to functional modifications and repolarization of TAMs. Increased glycolysis, decreased FAO, and a reprogrammed TCA cycle promoted the repolarization of TAMs into acquiring the proinflammatory phenotype. Metabolites produced during metabolic reprogramming such as lactate, α-KG, and succinic acid, also regulated macrophage activation. Therefore, it is critical to understand the cross talk among the factors involved in metabolic alterations and macrophage function.

In recent years, radiotherapy and chemotherapy have shown good efficacy in the treatment of malignant tumours. However, radiotherapy and chemotherapy resistance remain great challenges to effective cancer treatment. Many studies have revealed that abnormal lipid metabolism is associated with resistance to radiotherapy and chemotherapy [187–189]. Moreover, CPT1A has been shown to be highly expressed in radioresistant cancer cells and can increase the FAO rate, while inhibition of fatty acid synthesis or targeting CPT1A attenuated radioresistance and decreased radiation-mediated ERK activation [190, 191]. Radiation promoted macrophage differentiation into different phenotypes in a dose-dependent manner. For example, high doses of irradiation (20 Gy) triggered macrophage polarization into the acquisition of an anti-inflammatory phenotype, whereas low-dose irradiation (2 Gy) skewed macrophages to an anti-tumour phenotype [192, 193]. Therefore, different doses may trigger different metabolic reprogramming processes. Thus, it may be important to explore the metabolic reprogramming of TAMs after treating them with different radiation doses.

## Data Availability

Not applicable.

## Abbreviations

LPS: Lipopolysaccharide
TME: Tumour microenvironment
TAMs: Tumour-associated macrophages
HIF-1α: Hypoxia inducible factor-1α
VEGF: Vascular endothelial growth factor
TF: Tissue factor
CSF-1: Colony-stimulating factor-1
PI3K: Phosphoinositide 3-kinase
TLR: Toll-like receptor
OXPHOS: Oxidative phosphorylation
COX: Cyclooxygenase
iNOS: Inducible nitric oxide synthase
Arg1: Arginase 1
FAO: Fatty acid oxidation
NSCLC: Non-small cell lung cancer
TNF-α: Tumour necrosis factor-alpha
AMPK: AMP-activated protein kinase
PPARγ: Peroxisome proliferator-activated receptor gamma
PFK2: 6-Phosphofructo-2-kinase/fructose-2,6-bisphosphatase
IL: Interleukin
NF-κB: Nuclear factor-kappa B
PGC-1β: PPARγ co-activator-1β
STAT6: Signal transducer and activator of transcription 6
PPP: Pentose phosphate pathway
CARKL: Carbohydrate kinase-like
SHPK: Sedoheptulose kinase
S7P: Sedoheptulose-7-phosphate
NADPH: Nicotinamide adenine dinucleotide phosphate
Ru5P: Ribulose-5-phosphate
ROS: Reactive oxygen species
TBI: Traumatic brain injury
NOX2: NADPH oxidase 2
IRF5: IFN regulatory factor 5
MAPK: Mitogen-activated protein kinase
PHD: Prolyl hydroxylase
HRE: Hypoxia response elements
PDPK1: 3-Phosphoinositide-dependent protein kinase 1
PGK1: Phosphoglycerate kinase 1
T: Threonine
GBM: Glioblastoma
EMT: Epithelial–mesenchymal transition
FoxO1: Forkhead box O1
M-CSF: Macrophage-CSF
SUCNR1: Succinate activates succinate receptor 1
mTORC1: Mechanistic target of rapamycin complex 1
TFEB: Transcription factor EB
PKM2: Pyruvate kinase M2
FSTL1: Follistatin-like protein 1
ACAT1: Cholesterol acyltransferase 1
EPA: Eicosapentaenoic acid
DHA: Docosahexaenoic acid
PGE2: Prostaglandin E2
LXR: Ligands of the liver X receptor
COX2: Cyclooxygenase 2
LTA4H: Leukotriene a4 hydrolase
ALOX5: Arachidonate 5-lipoxygenase
DHCR24: 24-Dehydrocholesterol reductase
5-LO: 5-Lipoxygenase
MerTK: Mer tyrosine kinase
cGAMP: Cyclic guanosine monophosphate–adenosine monophosphate
STING: Stimulator of interferon genes
SREBP1: Sterol regulatory element-binding protein 1
CpG: Cytosine–guanine dinucleotide
FAO: Fatty acid oxidation
TCA: Tricarboxylic acid
JAK1: Janus kinase 1
SHP1: Src homology 2 domain-containing protein tyrosine phosphatase 1
PPAR: Peroxisome proliferator-activated receptor
MCAD: Medium-chain acyl-CoA dehydrogenase
CCL2: C–C motif chemokine ligand 2
PGE2: Prostaglandin E2
MGLL: Monoacylglycerol lipase
BCKAs: Branched-chain ketoacids
BCAAs: Branched-chain amino acids
ODC: Ornithine decarboxylase
GI: Gastrointestinal
CAC: Colitis-associated carcinogenesis
ARG2: Arginase 2
AKT: Serine/threonine protein kinase
GSH: Glutathione
Zeb1: Zinc finger E-box binding homeobox 1
ER: Oestrogen receptor
SGLT1: Sodium/glucose co-transporter 1
MIF: Migration inhibitory factor
PDA: Pancreatic ductal adenocarcinoma
CB-2: Endocannabinoid receptor-2
ABHD5: AB-hydrolase containing 5
SRM: Spermidine synthase
RNA-seq: RNA-sequence
PERK: Protein kinase RNA-like endoplasmic reticulum kinase
α-KG: α-Ketoglutarate
PSTA1: Phosphoserine aminotransferase 1
MAT: Methionine adenosylmethionine
TIPE1: Tumour necrosis factor α-induced protein 8-like 1
CAR: Chimeric antigen receptor
CRS: Cytokine release syndrome

## References

[1] Ngambenjawong C, Gustafson HH, Pun SH (2017). Progress in tumor-associated macrophage (TAM)-targeted therapeutics. Adv Drug Deliv Rev.

[2] Takeuchi O, Akira S (2010). Pattern recognition receptors and inflammation. Cell.

[3] Orecchioni M, Ghosheh Y, Pramod AB (2019). macrophage polarization: different gene signatures in M1(LPS+) vs. classically and M2(LPS-) vs. alternatively activated macrophages. Front Immunol.

[4] Viola A, Munari F, Sanchez-Rodriguez R (2019). The metabolic signature of macrophage responses. Front Immunol.

[5] Roszer T (2015). Understanding the mysterious M2 macrophage through activation markers and effector mechanisms. Mediators Inflamm.

[6] Shapouri-Moghaddam A, Mohammadian S, Vazini H (2018). Macrophage plasticity, polarization, and function in health and disease. J Cell Physiol.

[7] Kang S, Kumanogoh A (2020). The spectrum of macrophage activation by immunometabolism. Int Immunol.

[8] Miller A, Nagy C, Knapp B (2017). Exploring metabolic configurations of single cells within complex tissue microenvironments. Cell Metab.

[9] Vitale I, Manic G, Coussens LM (2019). Macrophages and metabolism in the tumor microenvironment. Cell Metab.

[10] Pittet MJ, Michielin O, Migliorini D (2022). Clinical relevance of tumour-associated macrophages. Nat Rev Clin Oncol.

[11] Li S, Yu J, Huber A (2022). Metabolism drives macrophage heterogeneity in the tumor microenvironment. Cell Rep.

[12] Cassetta L, Pollard JW (2018). Targeting macrophages: therapeutic approaches in cancer. Nat Rev Drug Discov.

[13] Mills CD, Kincaid K, Alt JM (2000). M-1/M-2 macrophages and the Th1/Th2 paradigm. J Immunol.

[14] DeNardo DG, Barreto JB, Andreu P (2009). CD4(+) T cells regulate pulmonary metastasis of mammary carcinomas by enhancing protumor properties of macrophages. Cancer Cell.

[15] Yang H, Zhang Q, Xu M (2020). CCL2-CCR2 axis recruits tumor associated macrophages to induce immune evasion through PD-1 signaling in esophageal carcinogenesis. Mol Cancer.

[16] Dallavalasa S, Beeraka NM, Basavaraju CG (2021). The role of tumor associated macrophages (TAMs) in cancer progression, chemoresistance, angiogenesis and metastasis—current status. Curr Med Chem.

[17] Sugahara M, Tanaka S, Tanaka T (2020). Prolyl hydroxylase domain inhibitor protects against metabolic disorders and associated kidney disease in obese type 2 diabetic mice. J Am Soc Nephrol.

[18] Li H, Somiya M, Kuroda S (2021). Enhancing antibody-dependent cellular phagocytosis by Re-education of tumor-associated macrophages with resiquimod-encapsulated liposomes. Biomaterials.

[19] Feng Q, Ma X, Cheng K (2022). Engineered bacterial outer membrane vesicles as controllable two-way adaptors to activate macrophage phagocytosis for improved tumor immunotherapy. Adv Mater.

[20] Munir MT, Kay MK, Kang MH (2021). Tumor-associated macrophages as multifaceted regulators of breast tumor growth. Int J Mol Sci.

[21] Lu CS, Shiau AL, Su BH (2020). Oct4 promotes M2 macrophage polarization through upregulation of macrophage colony-stimulating factor in lung cancer. J Hematol Oncol.

[22] Shi B, Chu J, Huang T (2021). The scavenger receptor MARCO expressed by tumor-associated macrophages are highly associated with poor pancreatic cancer prognosis. Front Oncol.

[23] Forssell J, Oberg A, Henriksson ML (2007). High macrophage infiltration along the tumor front correlates with improved survival in colon cancer. Clin Cancer Res.

[24] Laoui D, Van Overmeire E, Di Conza G (2014). Tumor hypoxia does not drive differentiation of tumor-associated macrophages but rather fine-tunes the M2-like macrophage population. Cancer Res.

[25] Folkman J (1971). Tumor angiogenesis: therapeutic implications. N Engl J Med.

[26] Bluff JE, Menakuru SR, Cross SS (2009). Angiogenesis is associated with the onset of hyperplasia in human ductal breast disease. Br J Cancer.

[27] Guo L, Akahori H, Harari E (2018). CD163+ macrophages promote angiogenesis and vascular permeability accompanied by inflammation in atherosclerosis. J Clin Invest.

[28] Zhu C, Kros JM, Cheng C (2017). The contribution of tumor-associated macrophages in glioma neo-angiogenesis and implications for anti-angiogenic strategies. Neuro Oncol.

[29] Wyckoff J, Wang W, Lin EY (2004). A paracrine loop between tumor cells and macrophages is required for tumor cell migration in mammary tumors. Cancer Res.

[30] Wei C, Yang C, Wang S (2019). Crosstalk between cancer cells and tumor associated macrophages is required for mesenchymal circulating tumor cell-mediated colorectal cancer metastasis. Mol Cancer.

[31] Ries CH, Cannarile MA, Hoves S (2014). Targeting tumor-associated macrophages with anti-CSF-1R antibody reveals a strategy for cancer therapy. Cancer Cell.

[32] Stromnes IM, Burrack AL, Hulbert A (2019). Differential effects of depleting versus programming tumor-associated macrophages on engineered T cells in pancreatic ductal adenocarcinoma. Cancer Immunol Res.

[33] Cao X, Li B, Chen J (2021). Effect of cabazitaxel on macrophages improves CD47-targeted immunotherapy for triple-negative breast cancer. J Immunother Cancer.

[34] Li D, Zhang Q, Li L (2022). beta2-Microglobulin maintains glioblastoma stem cells and induces M2-like polarization of tumor-associated macrophages. Cancer Res.

[35] Rodell CB, Ahmed MS, Garris CS (2019). Development of adamantane-conjugated TLR7/8 agonists for supramolecular delivery and cancer immunotherapy. Theranostics.

[36] Zhang C, Liu J, Liang Y (2013). Tumour-associated mutant p53 drives the Warburg effect. Nat Commun.

[37] Huang J, Sun W, Wang Z (2022). FTO suppresses glycolysis and growth of papillary thyroid cancer via decreasing stability of APOE mRNA in an N6-methyladenosine-dependent manner. J Exp Clin Cancer Res.

[38] Warburg O (1956). On respiratory impairment in cancer cells. Science.

[39] Tseng WW, Wei AC (2022). Kinetic mathematical modeling of oxidative phosphorylation in cardiomyocyte mitochondria. Cells.

[40] Reid MA, Allen AE, Liu S (2018). Serine synthesis through PHGDH coordinates nucleotide levels by maintaining central carbon metabolism. Nat Commun.

[41] Ngo B, Kim E, Osorio-Vasquez V (2020). Limited environmental serine and glycine confer brain metastasis sensitivity to PHGDH inhibition. Cancer Discov.

[42] Tanner LB, Goglia AG, Wei MH (2018). Four key steps control glycolytic flux in mammalian cells. Cell Syst.

[43] Dang CV (2012). Links between metabolism and cancer. Genes Dev.

[44] DeBerardinis RJ, Chandel NS (2016). Fundamentals of cancer metabolism. Sci Adv.

[45] Pavlova NN, Thompson CB (2016). The emerging hallmarks of cancer metabolism. Cell Metab.

[46] Elia I, Haigis MC (2021). Metabolites and the tumour microenvironment: from cellular mechanisms to systemic metabolism. Nat Metab.

[47] Liu PS, Wang H, Li X (2017). alpha-ketoglutarate orchestrates macrophage activation through metabolic and epigenetic reprogramming. Nat Immunol.

[48] Lauby-Secretan B, Scoccianti C, Loomis D (2016). Body fatness and cancer-viewpoint of the IARC Working Group. N Engl J Med.

[49] Li R, Grimm SA, Mav D (2018). Transcriptome and DNA methylome analysis in a mouse model of diet-induced obesity predicts increased risk of colorectal cancer. Cell Rep.

[50] Li R, Grimm SA, Chrysovergis K (2014). Obesity, rather than diet, drives epigenomic alterations in colonic epithelium resembling cancer progression. Cell Metab.

[51] Ringel AE, Drijvers JM, Baker GJ (2020). Obesity shapes metabolism in the tumor microenvironment to suppress anti-tumor immunity. Cell.

[52] Sipe LM, Chaib M, Pingili AK (2020). Microbiome, bile acids, and obesity: How microbially modified metabolites shape anti-tumor immunity. Immunol Rev.

[53] Ma C, Han M, Heinrich B (2018). Gut microbiome-mediated bile acid metabolism regulates liver cancer via NKT cells. Science.

[54] Fischer K, Hoffmann P, Voelkl S (2007). Inhibitory effect of tumor cell-derived lactic acid on human T cells. Blood.

[55] Macintyre AN, Gerriets VA, Nichols AG (2014). The glucose transporter Glut1 is selectively essential for CD4 T cell activation and effector function. Cell Metab.

[56] Li W, Tanikawa T, Kryczek I (2018). Aerobic glycolysis controls myeloid-derived suppressor cells and tumor immunity via a specific cebpb isoform in triple-negative breast cancer. Cell Metab.

[57] Richtig G, Hoeller C, Wolf M (2018). Body mass index may predict the response to ipilimumab in metastatic melanoma: An observational multi-centre study. PLoS ONE.

[58] McQuade JL, Daniel CR, Hess KR (2018). Association of body-mass index and outcomes in patients with metastatic melanoma treated with targeted therapy, immunotherapy, or chemotherapy: a retrospective, multicohort analysis. Lancet Oncol.

[59] Cortellini A, Bersanelli M, Buti S (2019). A multicenter study of body mass index in cancer patients treated with anti-PD-1/PD-L1 immune checkpoint inhibitors: when overweight becomes favorable. J Immunother Cancer.

[60] Jeong H, Kim S, Hong BJ (2019). Tumor-associated macrophages enhance tumor hypoxia and aerobic glycolysis. Cancer Res.

[61] Rodriguez-Prados JC, Traves PG, Cuenca J (2010). Substrate fate in activated macrophages: a comparison between innate, classic, and alternative activation. J Immunol.

[62] O'Neill LA, Pearce EJ (2016). Immunometabolism governs dendritic cell and macrophage function. J Exp Med.

[63] Chen DP, Ning WR, Jiang ZZ (2019). Glycolytic activation of peritumoral monocytes fosters immune privilege via the PFKFB3-PD-L1 axis in human hepatocellular carcinoma. J Hepatol.

[64] Vats D, Mukundan L, Odegaard JI (2006). Oxidative metabolism and PGC-1beta attenuate macrophage-mediated inflammation. Cell Metab.

[65] Yu T, Gan S, Zhu Q (2019). Modulation of M2 macrophage polarization by the crosstalk between Stat6 and Trim24. Nat Commun.

[66] Haschemi A, Kosma P, Gille L (2012). The sedoheptulose kinase CARKL directs macrophage polarization through control of glucose metabolism. Cell Metab.

[67] Cai W, Cheng J, Zong S (2021). The glycolysis inhibitor 2-deoxyglucose ameliorates adjuvant-induced arthritis by regulating macrophage polarization in an AMPK-dependent manner. Mol Immunol.

[68] Patra KC, Hay N (2014). The pentose phosphate pathway and cancer. Trends Biochem Sci.

[69] Xu C, Yang H, Xiao Z (2021). Reduction-responsive dehydroepiandrosterone prodrug nanoparticles loaded with camptothecin for cancer therapy by enhancing oxidation therapy and cell replication inhibition. Int J Pharm.

[70] Zheng W, Umitsu M, Jagan I (2016). An interaction between scribble and the NADPH oxidase complex controls M1 macrophage polarization and function. Nat Cell Biol.

[71] Roux C, Jafari SM, Shinde R (2019). Reactive oxygen species modulate macrophage immunosuppressive phenotype through the up-regulation of PD-L1. Proc Natl Acad Sci U S A.

[72] Han W, Fessel JP, Sherrill T (2020). Enhanced expression of catalase in mitochondria modulates NF-kappaB-dependent lung inflammation through alteration of metabolic activity in macrophages. J Immunol.

[73] Wang J, Ma MW, Dhandapani KM (2017). Regulatory role of NADPH oxidase 2 in the polarization dynamics and neurotoxicity of microglia/macrophages after traumatic brain injury. Free Radic Biol Med.

[74] Wu Q, Allouch A, Paoletti A (2017). NOX2-dependent ATM kinase activation dictates pro-inflammatory macrophage phenotype and improves effectiveness to radiation therapy. Cell Death Differ.

[75] Tannahill GM, Curtis AM, Adamik J (2013). Succinate is an inflammatory signal that induces IL-1beta through HIF-1alpha. Nature.

[76] Murdoch C, Muthana M, Lewis CE (2005). Hypoxia regulates macrophage functions in inflammation. J Immunol.

[77] Wang YT, Trzeciak AJ, Rojas WS (2023). Metabolic adaptation supports enhanced macrophage efferocytosis in limited-oxygen environments. Cell Metab.

[78] Lu LG, Zhou ZL, Wang XY (2022). PD-L1 blockade liberates intrinsic antitumourigenic properties of glycolytic macrophages in hepatocellular carcinoma. Gut.

[79] Luo W, Semenza GL (2012). Emerging roles of PKM2 in cell metabolism and cancer progression. Trends Endocrinol Metab.

[80] Palsson-McDermott EM, Curtis AM, Goel G (2015). Pyruvate kinase M2 regulates Hif-1alpha activity and IL-1beta induction and is a critical determinant of the warburg effect in LPS-activated macrophages. Cell Metab.

[81] Rao J, Wang H, Ni M (2022). FSTL1 promotes liver fibrosis by reprogramming macrophage function through modulating the intracellular function of PKM2. Gut.

[82] Cheng Y, Feng Y, Xia Z (2017). omega-Alkynyl arachidonic acid promotes anti-inflammatory macrophage M2 polarization against acute myocardial infarction via regulating the cross-talk between PKM2, HIF-1alpha and iNOS. Biochim Biophys Acta Mol Cell Biol Lipids.

[83] Zhang Y, Yu G, Chu H (2018). Macrophage-Associated PGK1 Phosphorylation Promotes Aerobic Glycolysis and Tumorigenesis. Mol Cell.

[84] Zhang J, Zhang Q, Lou Y (2018). Hypoxia-inducible factor-1alpha/interleukin-1beta signaling enhances hepatoma epithelial-mesenchymal transition through macrophages in a hypoxic-inflammatory microenvironment. Hepatology.

[85] Xiao P, Long X, Zhang L (2018). Neurotensin/IL-8 pathway orchestrates local inflammatory response and tumor invasion by inducing M2 polarization of tumor-associated macrophages and epithelial-mesenchymal transition of hepatocellular carcinoma cells. Oncoimmunology.

[86] Yang JB, Zhao ZB, Liu QZ (2018). FoxO1 is a regulator of MHC-II expression and anti-tumor effect of tumor-associated macrophages. Oncogene.

[87] El-Kenawi A, Gatenbee C, Robertson-Tessi M (2019). Acidity promotes tumour progression by altering macrophage phenotype in prostate cancer. Br J Cancer.

[88] Paolini L, Adam C, Beauvillain C (2020). Lactic acidosis together with GM-CSF and M-CSF induces human macrophages toward an inflammatory protumor phenotype. Cancer Immunol Res.

[89] Wu JY, Huang TW, Hsieh YT (2020). Cancer-derived succinate promotes macrophage polarization and cancer metastasis via succinate receptor. Mol Cell.

[90] Liu N, Luo J, Kuang D (2019). Lactate inhibits ATP6V0d2 expression in tumor-associated macrophages to promote HIF-2alpha-mediated tumor progression. J Clin Invest.

[91] Zhang D, Tang Z, Huang H (2019). Metabolic regulation of gene expression by histone lactylation. Nature.

[92] Casazza A, Laoui D, Wenes M (2013). Impeding macrophage entry into hypoxic tumor areas by Sema3A/Nrp1 signaling blockade inhibits angiogenesis and restores antitumor immunity. Cancer Cell.

[93] Wenes M, Shang M, Di Matteo M (2016). Macrophage metabolism controls tumor blood vessel morphogenesis and metastasis. Cell Metab.

[94] Yan J, Horng T (2020). Lipid metabolism in regulation of macrophage functions. Trends Cell Biol.

[95] Marelli G, Morina N, Portale F (2022). Lipid-loaded macrophages as new therapeutic target in cancer. J Immunother Cancer.

[96] Jaitin DA, Adlung L, Thaiss CA (2019). Lipid-associated macrophages control metabolic homeostasis in a Trem2-dependent manner. Cell.

[97] Di Gioia M, Spreafico R, Springstead JR (2020). Endogenous oxidized phospholipids reprogram cellular metabolism and boost hyperinflammation. Nat Immunol.

[98] An L, Lu M, Xu W (2022). Qingfei oral liquid alleviates RSV-induced lung inflammation by promoting fatty-acid-dependent M1/M2 macrophage polarization via the Akt signaling pathway. J Ethnopharmacol.

[99] Liu S, Zhang H, Li Y (2021). S100A4 enhances protumor macrophage polarization by control of PPAR-gamma-dependent induction of fatty acid oxidation. J Immunother Cancer.

[100] Ringleb J, Strack E, Angioni C (2018). Apoptotic cancer cells suppress 5-lipoxygenase in tumor-associated macrophages. J Immunol.

[101] Zhou Y, Fei M, Zhang G (2020). Blockade of the phagocytic receptor MerTK on tumor-associated macrophages enhances P2X7R-dependent STING activation by tumor-derived cGAMP. Immunity.

[102] Liu C, Chikina M, Deshpande R (2019). Treg cells promote the SREBP1-dependent metabolic fitness of tumor-promoting macrophages via repression of CD8(+) T cell-derived interferon-gamma. Immunity.

[103] Liu M, O'Connor RS, Trefely S (2019). Metabolic rewiring of macrophages by CpG potentiates clearance of cancer cells and overcomes tumor-expressed CD47-mediated 'don't-eat-me' signal. Nat Immunol.

[104] Su P, Wang Q, Bi E (2020). Enhanced lipid accumulation and metabolism are required for the differentiation and activation of tumor-associated macrophages. Cancer Res.

[105] Su P, Wang Q, Bi E (2020). Enhanced lipid accumulation and metabolism are required for the differentiation and activation of tumor-associated macrophages. Cancer Res.

[106] Montaigne D, Butruille L, Staels B (2021). PPAR control of metabolism and cardiovascular functions. Nat Rev Cardiol.

[107] Goossens P, Rodriguez-Vita J, Etzerodt A (2019). Membrane cholesterol efflux drives tumor-associated macrophage reprogramming and tumor progression. Cell Metab.

[108] Caputa G, Castoldi A, Pearce EJ (2019). Metabolic adaptations of tissue-resident immune cells. Nat Immunol.

[109] Varga T, Czimmerer Z, Nagy L (2011). PPARs are a unique set of fatty acid regulated transcription factors controlling both lipid metabolism and inflammation. Biochim Biophys Acta.

[110] Niu Z, Shi Q, Zhang W (2017). Caspase-1 cleaves PPARgamma for potentiating the pro-tumor action of TAMs. Nat Commun.

[111] Huang G, Yin L, Lan J (2018). Synergy between peroxisome proliferator-activated receptor gamma agonist and radiotherapy in cancer. Cancer Sci.

[112] Xu M, Wang X, Li Y (2021). Arachidonic acid metabolism controls macrophage alternative activation through regulating oxidative phosphorylation in PPARgamma dependent manner. Front Immunol.

[113] Xiang W, Shi R, Kang X (2018). Monoacylglycerol lipase regulates cannabinoid receptor 2-dependent macrophage activation and cancer progression. Nat Commun.

[114] Sica A, Bleve A, Garassino MC (2019). Membrane cholesterol regulates macrophage plasticity in cancer. Cell Metab.

[115] Silva LS, Poschet G, Nonnenmacher Y (2017). Branched-chain ketoacids secreted by glioblastoma cells via MCT1 modulate macrophage phenotype. EMBO Rep.

[116] Yoon BR, Oh YJ, Kang SW (2018). Role of SLC7A5 in metabolic reprogramming of human monocyte/macrophage immune responses. Front Immunol.

[117] Singh K, Coburn LA, Asim M (2018). Ornithine decarboxylase in macrophages exacerbates colitis and promotes colitis-associated colon carcinogenesis by impairing M1 immune responses. Cancer Res.

[118] Fultang L, Gamble LD, Gneo L (2019). Macrophage-derived IL1beta and TNFalpha regulate arginine metabolism in neuroblastoma. Cancer Res.

[119] Rodriguez AE, Ducker GS, Billingham LK (2019). Serine metabolism supports macrophage IL-1beta production. Cell Metab.

[120] Mills E, O'Neill LA (2014). Succinate: a metabolic signal in inflammation. Trends Cell Biol.

[121] Strelko CL, Lu W, Dufort FJ (2011). Itaconic acid is a mammalian metabolite induced during macrophage activation. J Am Chem Soc.

[122] Michelucci A, Cordes T, Ghelfi J (2013). Immune-responsive gene 1 protein links metabolism to immunity by catalyzing itaconic acid production. Proc Natl Acad Sci U S A.

[123] Lampropoulou V, Sergushichev A, Bambouskova M (2016). Itaconate links inhibition of succinate dehydrogenase with macrophage metabolic remodeling and regulation of inflammation. Cell Metab.

[124] Hooftman A, Angiari S, Hester S (2020). The immunomodulatory metabolite itaconate modifies NLRP3 and inhibits inflammasome activation. Cell Metab.

[125] Hoyle C, Green JP, Allan SM (2022). Itaconate and fumarate derivatives inhibit priming and activation of the canonical NLRP3 inflammasome in macrophages. Immunology.

[126] Mills EL, Ryan DG, Prag HA (2018). Itaconate is an anti-inflammatory metabolite that activates Nrf2 via alkylation of KEAP1. Nature.

[127] Ryan DG, Knatko EV, Casey AM (2022). Nrf2 activation reprograms macrophage intermediary metabolism and suppresses the type I interferon response. iScience.

[128] Bambouskova M, Gorvel L, Lampropoulou V (2018). Electrophilic properties of itaconate and derivatives regulate the IkappaBzeta-ATF3 inflammatory axis. Nature.

[129] Liao ST, Han C, Xu DQ (2019). 4-Octyl itaconate inhibits aerobic glycolysis by targeting GAPDH to exert anti-inflammatory effects. Nat Commun.

[130] Runtsch MC, Angiari S, Hooftman A (2022). Itaconate and itaconate derivatives target JAK1 to suppress alternative activation of macrophages. Cell Metab.

[131] Weiss JM, Davies LC, Karwan M (2018). Itaconic acid mediates crosstalk between macrophage metabolism and peritoneal tumors. J Clin Invest.

[132] Scheurlen KM, Snook DL, Walter MN (2022). Itaconate and leptin affecting PPARgamma in M2 macrophages: a potential link to early-onset colorectal cancer. Surgery.

[133] Zheng B (2022). Suppression of CD8 (+) T cells by the metabolite itaconate. Nat Metab.

[134] Krell D, Mulholland P, Frampton AE (2013). IDH mutations in tumorigenesis and their potential role as novel therapeutic targets. Future Oncol.

[135] Ming-Chin Lee K, Achuthan AA, De Souza DP (2022). Type I interferon antagonism of the JMJD3-IRF4 pathway modulates macrophage activation and polarization. Cell Rep.

[136] Zhou W, Hu G, He J (2022). SENP1-Sirt3 signaling promotes alpha-ketoglutarate production during M2 macrophage polarization. Cell Rep.

[137] Noe JT, Mitchell RA (2019). Tricarboxylic acid cycle metabolites in the control of macrophage activation and effector phenotypes. J Leukoc Biol.

[138] Li Y, Li YC, Liu XT (2022). Blockage of citrate export prevents TCA cycle fragmentation via Irg1 inactivation. Cell Rep.

[139] Infantino V, Iacobazzi V, Menga A (2014). A key role of the mitochondrial citrate carrier (SLC25A1) in TNFalpha- and IFNgamma-triggered inflammation. Biochim Biophys Acta.

[140] Covarrubias AJ, Aksoylar HI, Yu J (2016). Akt-mTORC1 signaling regulates Acly to integrate metabolic input to control of macrophage activation. Elife.

[141] Gomez-Roca CA, Italiano A, Le Tourneau C (2019). Phase I study of emactuzumab single agent or in combination with paclitaxel in patients with advanced/metastatic solid tumors reveals depletion of immunosuppressive M2-like macrophages. Annals Oncol.

[142] Kamerkar S, Leng C, Burenkova O (2022). Exosome-mediated genetic reprogramming of tumor-associated macrophages by exoASO-STAT6 leads to potent monotherapy antitumor activity. Sci Adv.

[143] Hiratsuka S, Watanabe A, Sakurai Y (2008). The S100A8-serum amyloid A3-TLR4 paracrine cascade establishes a pre-metastatic phase. Nat Cell Biol.

[144] Peinado H, Aleckovic M, Lavotshkin S (2012). Melanoma exosomes educate bone marrow progenitor cells toward a pro-metastatic phenotype through MET. Nat Med.

[145] Olkhanud PB, Damdinsuren B, Bodogai M (2011). Tumor-evoked regulatory B cells promote breast cancer metastasis by converting resting CD4(+) T cells to T-regulatory cells. Cancer Res.

[146] Fong MY, Zhou W, Liu L (2015). Breast-cancer-secreted miR-122 reprograms glucose metabolism in premetastatic niche to promote metastasis. Nat Cell Biol.

[147] Headley MB, Bins A, Nip A (2016). Visualization of immediate immune responses to pioneer metastatic cells in the lung. Nature.

[148] Linde N, Casanova-Acebes M, Sosa MS (2018). Macrophages orchestrate breast cancer early dissemination and metastasis. Nat Commun.

[149] Morrissey SM, Zhang F, Ding C (2021). Tumor-derived exosomes drive immunosuppressive macrophages in a pre-metastatic niche through glycolytic dominant metabolic reprogramming. Cell Metab.

[150] Jiang H, Wei H, Wang H (2022). Zeb1-induced metabolic reprogramming of glycolysis is essential for macrophage polarization in breast cancer. Cell Death Dis.

[151] Castellaro AM, Rodriguez-Baili MC, Di Tada CE (2019). Tumor-associated macrophages induce endocrine therapy resistance in ER+ breast cancer cells. Cancers.

[152] Niu X, Ma J, Li J (2021). Sodium/glucose cotransporter 1-dependent metabolic alterations induce tamoxifen resistance in breast cancer by promoting macrophage M2 polarization. Cell Death Dis.

[153] Penault-Llorca F, Viale G (2012). Pathological and molecular diagnosis of triple-negative breast cancer: a clinical perspective. Ann Oncol.

[154] Yang X, Zhao M, Wu Z (2022). Nano-ultrasonic contrast agent for chemoimmunotherapy of breast cancer by immune metabolism reprogramming and tumor autophagy. ACS Nano.

[155] Tian LR, Lin MZ, Zhong HH (2022). Nanodrug regulates lactic acid metabolism to reprogram the immunosuppressive tumor microenvironment for enhanced cancer immunotherapy. Biomater Sci.

[156] Figueiredo CR, Azevedo RA, Mousdell S (2018). Blockade of MIF-CD74 signalling on macrophages and dendritic cells restores the antitumour immune response against metastatic melanoma. Front Immunol.

[157] Tanese K, Hashimoto Y, Berkova Z (2015). Cell surface CD74-MIF interactions drive melanoma survival in response to interferon-gamma. J Invest Dermatol.

[158] de Azevedo RA, Shoshan E, Whang S (2020). MIF inhibition as a strategy for overcoming resistance to immune checkpoint blockade therapy in melanoma. Oncoimmunology.

[159] Jiang Y, Han Q, Zhao H (2021). Promotion of epithelial-mesenchymal transformation by hepatocellular carcinoma-educated macrophages through Wnt2b/beta-catenin/c-Myc signaling and reprogramming glycolysis. J Exp Clin Cancer Res.

[160] Shan M, Qin J, Jin F (2017). Autophagy suppresses isoprenaline-induced M2 macrophage polarization via the ROS/ERK and mTOR signaling pathway. Free Radic Biol Med.

[161] Gomez-Martin C, Bustamante J, Castroagudin JF (2012). Efficacy and safety of sorafenib in combination with mammalian target of rapamycin inhibitors for recurrent hepatocellular carcinoma after liver transplantation. Liver Transpl.

[162] Chen B, Gao A, Tu B (2020). Metabolic modulation via mTOR pathway and anti-angiogenesis remodels tumor microenvironment using PD-L1-targeting codelivery. Biomaterials.

[163] Zhang M, Pan X, Fujiwara K (2021). Pancreatic cancer cells render tumor-associated macrophages metabolically reprogrammed by a GARP and DNA methylation-mediated mechanism. Signal Transduct Target Ther.

[164] Cao S, Saw PE, Shen Q (2022). Reduction-responsive RNAi nanoplatform to reprogram tumor lipid metabolism and repolarize macrophage for combination pancreatic cancer therapy. Biomaterials.

[165] Lass A, Zimmermann R, Haemmerle G (2006). Adipose triglyceride lipase-mediated lipolysis of cellular fat stores is activated by CGI-58 and defective in Chanarin–Dorfman syndrome. Cell Metab.

[166] Miao H, Ou J, Peng Y (2016). Macrophage ABHD5 promotes colorectal cancer growth by suppressing spermidine production by SRM. Nat Commun.

[167] Raines LN, Zhao H, Wang Y (2022). PERK is a critical metabolic hub for immunosuppressive function in macrophages. Nat Immunol.

[168] Ahirwar DK, Charan M, Mishra S (2021). Slit2 inhibits breast cancer metastasis by activating M1-like phagocytic and antifibrotic macrophages. Cancer Res.

[169] Kaul K, Benej M, Mishra S (2021). Slit2-mediated metabolic reprogramming in bone marrow-derived macrophages enhances antitumor immunity. Front Immunol.

[170] Ramesh A, Malik V, Brouillard A (2022). Supramolecular nanotherapeutics enable metabolic reprogramming of tumor-associated macrophages to inhibit tumor growth. J Biomed Mater Res A.

[171] Yang Z, Wang Y, Zhang Y (2018). RIP3 targets pyruvate dehydrogenase complex to increase aerobic respiration in TNF-induced necroptosis. Nat Cell Biol.

[172] Wu L, Zhang X, Zheng L (2020). RIPK3 orchestrates fatty acid metabolism in tumor-associated macrophages and hepatocarcinogenesis. Cancer Immunol Res.

[173] Ji L, Zhao X, Zhang B (2019). Slc6a8-mediated creatine uptake and accumulation reprogram macrophage polarization via regulating cytokine responses. Immunity.

[174] Wang Z, Yip LY, Lee JHJ (2019). Methionine is a metabolic dependency of tumor-initiating cells. Nat Med.

[175] Zhang T, Zheng Z, Liu Y (2013). Overexpression of methionine adenosyltransferase II alpha (MAT2A) in gastric cancer and induction of cell cycle arrest and apoptosis in SGC-7901 cells by shRNA-mediated silencing of MAT2A gene. Acta Histochem.

[176] Zhang Y, Yang H, Zhao J (2021). Activation of MAT2A-RIP1 signaling axis reprograms monocytes in gastric cancer. J Immunother Cancer.

[177] Czech MP (2000). PIP2 and PIP3: complex roles at the cell surface. Cell.

[178] Di Paolo G, De Camilli P (2006). Phosphoinositides in cell regulation and membrane dynamics. Nature.

[179] Cheng Y, Bai F, Ren X (2022). Phosphoinositide-binding protein TIPE1 promotes alternative activation of macrophages and tumor progression via PIP3/Akt/TGFbeta axis. Cancer Res.

[180] Cercek A, Lumish M, Sinopoli J (2022). PD-1 blockade in mismatch repair-deficient, locally advanced rectal cancer. N Engl J Med.

[181] Cloughesy TF, Mochizuki AY, Orpilla JR (2019). Neoadjuvant anti-PD-1 immunotherapy promotes a survival benefit with intratumoral and systemic immune responses in recurrent glioblastoma. Nat Med.

[182] Gao S, Li N, Gao S (2020). Neoadjuvant PD-1 inhibitor (Sintilimab) in NSCLC. J Thorac Oncol.

[183] Neelapu SS, Locke FL, Bartlett NL (2017). Axicabtagene ciloleucel CAR T-cell therapy in refractory large B-cell lymphoma. N Engl J Med.

[184] Ramos CA, Grover NS, Beaven AW (2020). Anti-CD30 CAR-T cell therapy in relapsed and refractory hodgkin lymphoma. J Clin Oncol.

[185] Giavridis T, van der Stegen SJC, Eyquem J (2018). CAR T cell-induced cytokine release syndrome is mediated by macrophages and abated by IL-1 blockade. Nat Med.

[186] Xu Y, Zeng H, Jin K (2022). Immunosuppressive tumor-associated macrophages expressing interlukin-10 conferred poor prognosis and therapeutic vulnerability in patients with muscle-invasive bladder cancer. J Immunother Cancer.

[187] Tan Z, Xiao L, Tang M (2018). Targeting CPT1A-mediated fatty acid oxidation sensitizes nasopharyngeal carcinoma to radiation therapy. Theranostics.

[188] Koppula P, Lei G, Zhang Y (2022). A targetable CoQ-FSP1 axis drives ferroptosis- and radiation-resistance in KEAP1 inactive lung cancers. Nat Commun.

[189] Jin H, He Y, Zhao P (2019). Targeting lipid metabolism to overcome EMT-associated drug resistance via integrin beta3/FAK pathway and tumor-associated macrophage repolarization using legumain-activatable delivery. Theranostics.

[190] Han S, Wei R, Zhang X (2019). CPT1A/2-mediated FAO enhancement-A metabolic target in radioresistant breast cancer. Front Oncol.

[191] Du Q, Tan Z, Shi F (2019). PGC1alpha/CEBPB/CPT1A axis promotes radiation resistance of nasopharyngeal carcinoma through activating fatty acid oxidation. Cancer Sci.

[192] Nadella V, Singh S, Jain A (2018). Low dose radiation primed iNOS + M1macrophages modulate angiogenic programming of tumor derived endothelium. Mol Carcinog.

[193] Klug F, Prakash H, Huber PE (2013). Low-dose irradiation programs macrophage differentiation to an iNOS(+)/M1 phenotype that orchestrates effective T cell immunotherapy. Cancer Cell.

